# Application of PLCL as a biodegradable polymer in biomedical engineering

**DOI:** 10.1007/s10856-026-07016-3

**Published:** 2026-02-27

**Authors:** Yonggang Zhao, Honglei Liu

**Affiliations:** 1 Environment Monitoring Center of Jiangsu Province, Nanjing, China; 2 Nanjing Shenghong Environmental Technology Co. Ltd, Nanjing, China

## Abstract

Poly (L-lactide-co-ε-caprolactone) (PLCL) is a novel polymer that has attracted considerable attention in the biomedical field due to its exceptional biocompatibility. However, a comprehensive and systematic summary of its diverse applications remains lacking. To address this gap, the present review outlines the physicochemical properties of PLCL and the factors that influence them. Additionally, it consolidates the most commonly employed processing and preparation methods for PLCL in biomedical applications. The review further provides a systematic overview of current applications of PLCL in various biomedical fields, including wound healing, cardiovascular stents, nerve repair, osteochondral tissue engineering, drug delivery, and screening. It also examines modification strategies aimed at enhancing PLCL performance. Ultimately, this review seeks to provide valuable insights for future research and development of PLCL in biomedical contexts.

**Graphical Abstract:**

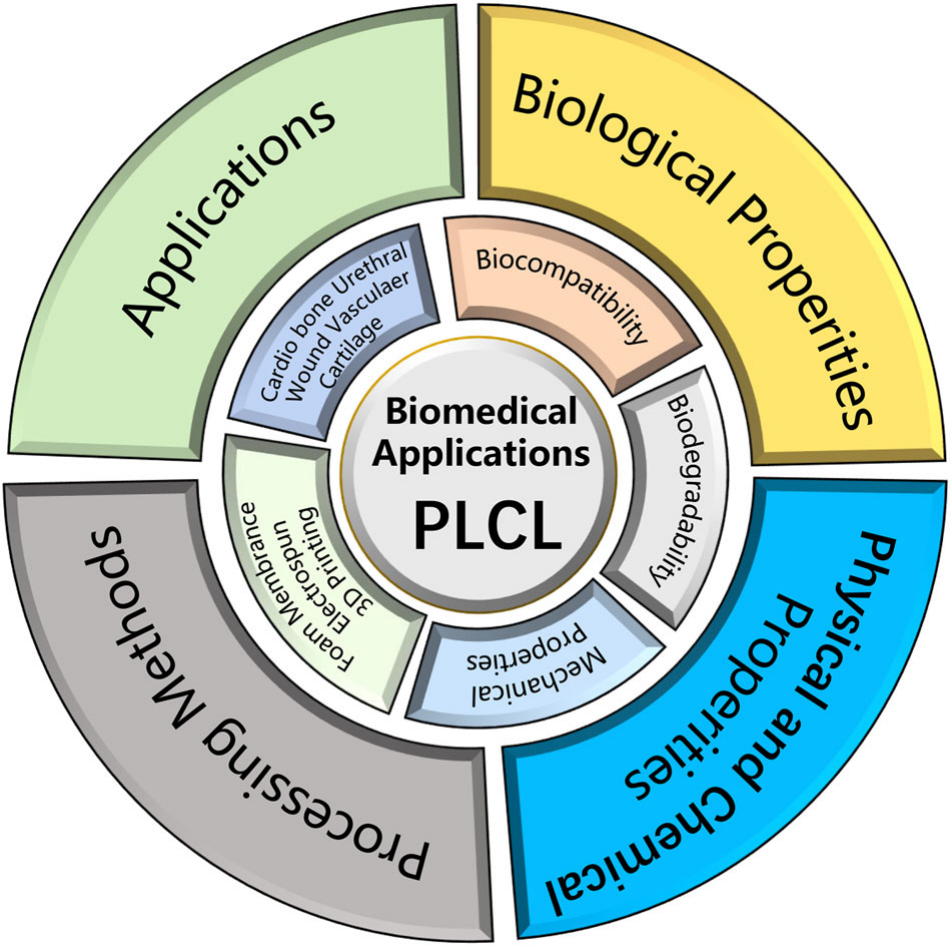

## Introduction

Poly (L-lactide-co-ε-caprolactone) (PLCL) has become an important biomaterial in biomedical engineering due to its excellent biocompatibility, tunable biodegradability, and multifunctional mechanical properties [1–4]. As a copolymer synthesized from poly (L-lactic acid) (PLLA) and poly (ε-caprolactone) (PCL), PLCL combines the rigidity and structural integrity of PLLA with the flexibility and elasticity of PCL, making it particularly suitable for a wide range of tissue engineering and regenerative medicine applications [5–8]. Advances in polymer science have allowed precise control of the monomer and molecular weight ratios during PLCL synthesis, enabling customization of its degradation rate, mechanical strength, and cellular interactions to meet specific biomedical needs [9, 10]. This tunability is essential for addressing challenges such as achieving appropriate support and degradation times for different tissue types, ensuring integration with native tissues, and promoting cell proliferation and differentiation.

In addition to its inherent properties, PLCL’s adaptability through surface modification, blending with bioactive molecules, and processing through advanced manufacturing techniques such as electrospinning, 3D printing, and supercritical carbon dioxide (scCO₂) foaming has extended its use to multiple fields [11–13]. These applications include wound healing, vascular transplantation, bone and cartilage repair, and even drug delivery systems [14–20]. The customized properties of PLCL and the synergistic benefits of innovative processing methods make it a cornerstone material for the development of next-generation biomedical devices. We use Web of Science as the search engine for literature retrieval. First, we entered the keyword “PLCL” to define the study scope of the review. Within these identified literatures, we then conducted searches using the keywords “Electrospinning/coaxial electrospinning”, “3D printing”, “scCO₂”, “Membranes”, and “Aerogel scaffolds” respectively, to summarize the methods of preparing scaffolds using PLCL. Subsequently, in the same set of literature, we searched with the keywords “Wound healing”, “Vascular tissue engineering”, “Cardiovascular tissue engineering”, “Nerve tissue engineering”, “Cartilage tissue engineering”, “Bone tissue engineering”, and “Urethral tissue engineering” to classify and summarize the literature based on different application fields. This review aims to fully explore the multifaceted properties of PLCL, highlight the latest advances in its processing technology, and discuss its applications in tissue engineering to gain insights into its potential to transform modern medicine.

## Physical and chemical properties of PLCL

### Biocompatibility

Biocompatibility refers to the ability of materials to coexist harmoniously with cells and tissues in the body when they interact with biological tissues or body fluids without inducing rejection, inflammation, allergic reactions or toxicity [21]. This requires not only good biocompatibility of the material itself, but also that the material will not produce harmful degradation products during the degradation process [22]. Biological tissue engineering materials based on PLCL can effectively promote the adhesion and proliferation of a variety of cell types, such as fibroblasts, endothelial cells, and osteocytes [23, 24]. PLCL surface modification or covalent binding of bioactive molecules, such as cell adhesion factors and cell growth factors, can further improve their compatibility with specific cells [25–27]. For example, in neural tissue engineering, when PLCL is combined with neurotrophic factors, it can significantly improve nerve cell adhesion and axonal extension, and promote the repair of neural tissue [28].

The degradation products of PLCL are mainly L-lactate and ε-caprolactone, both of which are metabolic intermediates in the normal metabolic pathways of the organism. L-lactate can be metabolized to carbon dioxide and water by the citric acid cycle, whereas caprolactone can be metabolized to water and carbon dioxide by β-oxidation or excreted in the urine [29, 30]. Therefore, PLCL materials usually do not trigger severe immune responses or chronic inflammatory responses when degraded in vivo. Both in vitro and in vivo experiments show that PLCL maintains good histocompatibility after implantation, usually only triggers a mild, acute local inflammatory response, which is part of the normal wound healing process and is self-limiting within a short period of time [31, 32]. At the same time, the long-term existence of PLCL in vivo did not produce toxic accumulation in other organs [33]. The good biocompatibility of PLCL also ensures its wide application in the design of many biological tissue engineering materials.

### Biodegradability

When biomaterials are applied in living organisms, they often serve as bridges or carriers in the early stage, such as tissue engineering scaffolds or drug delivery systems, to provide support and protection for damaged local bodies, and further deliver cells, drugs and other factors to promote tissue regeneration [34, 35]. With the continuous regeneration and repair of the body and tissues, biological materials need to be gradually metabolized and degraded in the body, thus providing space for tissue regeneration and regrowth [36]. Therefore, biological materials need to have a good degradation rate when applied in vivo.

PLCL degrades over time in the physiological environment through hydrolytic cleavage of ester bonds, and its degradation rate can be adjusted by changing the ratio of L-lactide to ε-caprolactone and by changing the molecular weight of the polymer [37, 38]. The rigid molecular skeleton of L-lactide makes the material more susceptible to hydrolysis reactions, and higher L-lactide content usually accelerates the degradation process [39]. In contrast, since caprolactone has a longer aliphatic chain and is not easily hydrolyzed by hydrolytic enzymes, a higher ε-caprolactone content would slow down the degradation process, thus allowing the biomaterial to remain stable in the body for a longer time [40, 41]. In addition, the molecular weight of PLCL will also have an effect on the degradation time of PLCL. PLCL used in biomaterials can be roughly categorized into low-molecular-weight (20,000–50,000 Da), medium-molecular-weight (50,000–100,000 Da), and high-molecular-weight (100,000–300,000 Da) types based on their molecular weight. Low-molecular-weight PLCL materials have shorter chain segments, so they are more susceptible to hydrolysis and degradation within a shorter timeframe. PLCL with a higher molecular weight contains longer polymer chain segment structures, so more hydrolysis steps are needed to break the polymer backbone during degradation, and the degradation time is correspondingly longer [37]. By adjusting the ratio of L-lactide to ε-caprolactone and the molecular weight of the synthesized PLCL, the degradation time of PLCL can be flexibly adjusted from weeks to years [42]. For example, in the design of drug delivery systems or skin wound repair materials, a faster degradation rate is often required, and a higher L-lactide ratio can be used to synthesize low molecular weight PLCL [43]. However, when applied in bone, cartilage or vascular tissue engineering scaffolds, the process of tissue regeneration usually takes from a few months to a few years due to the prolonged regeneration process. In this case, a higher proportion of caprolactone and a higher molecular weight of PLCL could provide a longer degradation time to support the long-term tissue regeneration process [44].

In addition to PLCL molecular weight and monomer ratio, external factors can also affect the degradation time of PLCL. The temperature, pH, and moisture content of the implantation site also affect the degradation rate of PLCL. Increasing temperature accelerates the degradation of PLCL, and in vivo (37 °C) generally increases the rate of PLCL degradation significantly compared to room temperature (20–25 °C) [41, 45]. In acidic environments, the degradation rate of PLCL is usually faster. This is due to acidic conditions that increase the hydrolysis of ester bonds in the polymer chain. However, alkaline conditions will slow the degradation of the material [46, 47]. Therefore, the impact of the specific application environment on the degradation rate should also be considered in the material design. For example, PLCL biomaterials used in deep tissues (e.g., vascular stents) tend to degrade faster than those used on the body surface (e.g., wound dressings) [48]. For areas with inflammation or infection, which may induce an acidic microenvironment locally, PLCL degradation may be accelerated, requiring corresponding adjustments based on actual conditions [49].

### Mechanical properties

The mechanical properties of PLCL mainly include tensile strength, elastic modulus, elongation at break, and fatigue strength. These properties are not only related to its molecular weight, monomer ratio, and processing techniques, but also affected by external environmental factors such as temperature and humidity [38, 50, 51]. Tensile strength is an important index to measure the ability of materials to resist tensile force. In PLCL, the tensile strength is closely related to the ratio of L-lactic acid to caprolactone. The rigid structure of L-lactide monomers enables PLCL to exhibit higher tensile strength at higher L-lactide contents [52, 53]. Typically, the tensile strength is between 20 and 60 MPa, depending on its molecular weight and proportion. For example, a higher proportion of L-lactic acid often results in increased tensile strength and is suitable for applications requiring higher mechanical strength, such as bone tissue engineering scaffolds [54].

Elastic modulus reflects the deformation ability of a material under external force and is a key parameter to evaluate the rigidity of a material. The elastic modulus of PLCL generally ranges from 400 to 600 MPa, and this depends on its molecular weight, polymer structure and composition ratio [55]. High molecular weight PLCL generally exhibits a higher elastic modulus, which allows it to maintain better shape stability under loading, and is suitable for applications subjected to heavy loads [56]. Because PLCL has an adjustable modulus of elasticity, researchers can design materials of varying rigidity by varying the proportion of monomers to suit a variety of biomedical needs.

Elongation at break is an important index to measure the plasticity of materials, indicating the deformation ability of materials before fracture. The elongation at break of PLCL is typically high, ranging from 100 to 500%, especially when the ε-caprolactone proportion is increased, as this significantly enhances the material’s flexibility [57, 58]. The high fracture elongation means that PLCL is able to withstand large deformation without fracture in applications, and is suitable for tissue engineering applications that require flexibility, such as soft tissue scaffolds and vascular repair materials [59, 60]. This superior flexibility allows PLCL to better adapt to the dynamic changes in the biological environment after implantation in the body.

In addition to the constituent ratio and molecular weight of PLCL itself, the factors affecting the mechanical properties of PLCL after in vivo implantation mainly include temperature, humidity, and processing technology [11, 39]. When the temperature increases, the tensile strength and elastic modulus of PLCL decrease, and the fatigue strength also decreases. This is because elevated temperatures increase the mobility of polymer chains and enhance the fluidity of molecular chains, which shows that the rigidity of the material is weakened, and it is more prone to deformation. However, PLCL has a higher fracture elongation when the temperature increases, and the flexibility and ductility of the material are improved [61]. In addition, humidity also has an effect on the mechanical properties of PLCL, and higher humidity accelerates PLCL degradation, thereby reducing its mechanical strength [62]. Finally, different processing techniques also affect the mechanical properties of PLCL. PLCL fibers prepared by electrospinning techniques usually have high tensile strength. This is because during electrospinning, fiber orientation is enhanced and a more ordered structure is formed [63]. The PLCL prepared by 3D printing can be controlled by adjusting the printing parameters, such as packing density and interlayer bonding, to impart higher elastic modulus and fatigue strength to the material, which is more suitable for long-term implantation scenarios [64]. PLCL materials fabricated via supercritical CO₂ foaming technology tend to have higher porosity and lower density. Higher specific surface area can improve the biocompatibility and cell adhesion of the materials, but the mechanical strength is also reduced [65]. Depending on the application scenario, PLCL materials with specific properties can be obtained by selecting the appropriate preparation process and material ratio to meet the diverse needs in biomedical engineering.

The differences in physicochemical properties between PLCL and other commonly used biomaterials (PLA, PCL, PLGA and PGA) are shown in Table 1. Compared with other biomaterials, PLCL exhibits core advantages including excellent biocompatibility and flexible tunability of degradation rate and mechanical properties, thereby enabling adaptability to diverse tissue engineering scenarios [66, 67]. Its degradation products are non-toxic and non-cumulative, inducing only mild inflammatory responses [5]. The main limitations are that its degradation rate is susceptible to environmental factors and its mechanical property stability depends on external conditions, which necessitate scenario-specific adjustments to material formulations and fabrication processes and increase the complexity of material design. In practical biomedical applications, appropriate biomaterials should be selected based on specific application directions.

**Table 1:** Comparison of basic physicochemical and mechanical properties of common biomaterials

	PLCL	PLA	PCL	PLGA	PGA
monomer	L-lactic acid, ε-caprolactone [37]	L-lactic acid [232]	ε-caprolactone [233]	L-lactic acid, glycolide [234]	glycolide [235]
Mw	10,000–700,000 [30, 51, 236, 237]	50,000– 250,000 [238, 239]	15,000 –100,000 [240, 241]	40,000– 200,000 [238, 242]	100,000– 2,000,000 [243, 244]
Tg	50–60 °C [51, 245]	50–64°C [246]	–60 °C [247]	45–55 °C [248]	35–45 °C [249]
Tm	160–180 °C [51, 250]	145–186 °C [246]	55–65 °C [247, 251]	190–210°C [252]	210–230 °C [253]
Tensile strength	20–60 MPa [52, 53]	50–70 MPa [59, 254]	15–30 MPa [58, 63, 255]	40–50 MPa [256]	50–60 MPa [257]
Elongation at break(%)	100–500% [60]	1–5% [254, 258]	1000–2000% [168]	100–200% [259]	3–10% [260]
Modulus of elasticity	400–600 MPa [55, 56]	2500–3500 MPa [62, 261]	50–100 MPa [262]	500–1000 MPa [263]	2000–3000 MPa [64]

## PLCL processing methods in biomedical engineering

The different processing methods of PLCL, as well as the characteristics and application fields of materials prepared by these methods, are shown in Table 2.

**Table 2:** PLCL processing method

Processing method	Characteristics	Application fields
Electrospinning/coaxial electrospinning	High surface area, three-dimensional network porous structure, excellent tensile properties, controllable morphology, drug loading, and drug sustained release	Wound repair [264], nerve regeneration [265], vascular graft [266], cartilage repair [17, 267, 268], bone defect [207, 269],
3D printing	Personalized customization, controllable macro- and micro-structures, high-precision structures, and rapid preparation	Vascular graft [155], cartilage repair, never regeneration [183, 270, 271], bone defect [271], urethral tissue engineering [211]
scCO₂	Green and environmentally friendly, mild processing conditions, high surface area, precise regulation of pore structure	Vascular graft [120], cartilage repair [118, 272], bone defect [13]
Membranes	Dense structure, transparency, multi-layer structure, elasticity and flexibility	Bone defect [118], corneal repair [216]
Aerogel scaffolds	High porosity and specific surface area, low density, excellent adsorption and loading capacity, adjustable mechanical properties, and excellent environmental stability	Wound repair [126], bone defect [273], anti-adhesion effect after cardiac surgery [274]

### Electrospun nanofiber

**Fig. 1:**
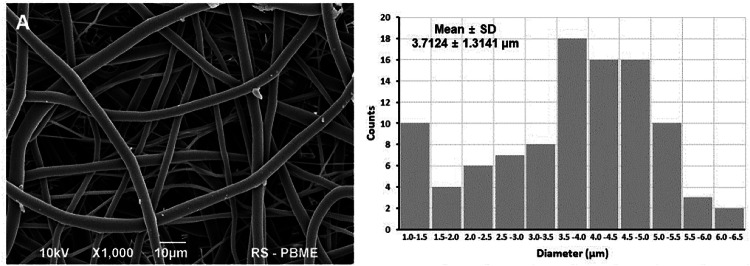
Morphology and diameter of PLCL nanofiber membranes [74]

Electrospinning is a technique that utilizes high-voltage electrostatic fields to stretch polymer solutions or melts into fibers. PLCL is dissolved in volatile organic solvents such as chloroform to form a PLCL solution. An electric field is established between the spinning nozzle and the collector through a high-voltage power supply [68, 69]. Under the combined action of the electric field force and electrostatic repulsion force, the PLCL droplets at the spinneret tip are stretched into a conical shape [70]. The fibers are ejected from the nozzle and gradually taper during the stretching process to form fibers deposited on the receiving plate, forming a nonwoven fabric or fibrous membrane with a specific structural orientation [71]. By adjusting the concentration and viscosity of the solution, the voltage of the applied electric field, the distance between the receiving plate and the emission nozzle, and the shape and type of the collector such as static or rotating, the electrospinning fibers with different fiber diameters, fiber porosity, specific surface area and spinning arrangements can be obtained [63, 72, 73]. As shown in Fig. 1, the SEM images exhibit the microtopography of a PLCL fiber membrane prepared *via* electrospinning technology [74]. The high specific surface area of electrospinning is conducive to cell attachment and growth, and the porous structure is conducive to the infiltration and exchange of nutrients [75–77]. Aligned electrospun fibers further impart directional cues to the material, improve the tensile strength of PLCL materials, and guide the directional growth of cells and tissues [78]. The above advantages make electrospinning technology especially suitable for the design and preparation of biomaterials for vascular repair and peripheral nerve repair [79, 80]. On this basis, bioactive substances such as anticoagulant factors, growth factors, and electroactive materials can be incorporated into PLCL to further improve the comprehensive performance of PLCL materials and make them more suitable for their application scenarios [81–83]. In recent years, a novel fiber fabrication technique, solution blow spinning (SBS), has been developed. Eliton S. Medeiros et al. systematically investigated the differences in structural and morphological characterization between fibers prepared by electrospinning and SBS techniques. The results showed that fibers fabricated *via* the SBS method exhibit minimal solvent residues, the fiber scaffolds had diameters ranging from the nanoscale to the microscale, and PLCL materials could be fabricated into various scaffolds *via* the SBS method for applications in diverse biomedical fields [84–87].

### 3D printing

3D printing technology has a wide range of applications in the preparation of PLCL biomaterials. 3D printing enables the fabrication of complex three-dimensional structures *via* computer-aided design, allows precise control over scaffold porosity, pore size, and microstructure, and facilitates accurate modulation of mechanical strength, and creates microstructures with graded porosity within a single scaffold [88–90]. This excellent property makes 3D printing technology suitable for the design of customized materials and materials with hierarchical structures, such as customized bone, cartilage, and joint prostheses, multilayer skin wound dressings, and vascular stent coatings [91].

**Fig. 2:**
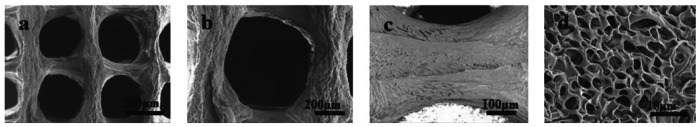
**a**–**d** images of PLCL scaffolds prepared by LTD method at different magnifications [108]

Common techniques for 3D printing PLCL scaffolds include fused deposition modelling (FDM), stereolithography (SLA), melt electrostatic writing (MEW), selective laser sintering (SLS) and low-temperature deposition 3D printing (LTD) [92–95]. Different preparation methods can be selected according to the required properties and different requirements for the material: FDM technology has lower requirements for the equipment, and the structure and strength of the PLCL scaffold can be controlled by adjusting the printing speed, temperature and layer thickness. However, this technique yields PLCL scaffolds with relatively low resolution, making it unsuitable for fabricating scaffolds with intricate structural requirements [96, 97]. The printing temperature of FDM is typically above the melting temperature of PLCL. The high-temperature printing process is not suitable for incorporating bioactive molecules that inactivate at high temperatures, thus imposing certain limitations on FDM printing [1]. SLA method is to construct a three-dimensional structure by layer-by-layer curing of photosensitive liquid resins using laser or ultraviolet light [98, 99]. This technique achieves high resolution and is suitable for fabricating intricate structures [100, 101]. Biodegradable polymers require the introduction of dimethacrylate (DMA) groups at their molecular terminals *via* chemical modification, and the modified materials can be used for SLA printing [102]. Photoinitiators need to be added to SLA printing inks, and residual photoinitiators may induce cytotoxicity, a key limitation of SLA printing [103]. MEW technology is a combination of electrospinning and 3D printing technology, by uses electrostatic stretching to accurately deposit PLCL melted fibers on the substrate, can prepare micron and sub-micron fiber structures, suitable for vascular grafts, nerves, skin and other scaffolds requiring high resolution and porous structure [104]. SLS technology is to sinter PLCL powder layer by layer with a laser to form a dense or porous scaffold structure. This method is suitable for the fabrication of scaffolds with complex geometric shapes, and is especially suitable for the construction of bone tissue scaffolds because of its high mechanical strength and porosity [105, 106]. The SLS method, like FDM, involves a high-temperature process, so it is also not suitable for preparing scaffolds using PLCL composites that contain temperature-sensitive bioactive molecules [107]. For the LTD method, first dissolve PLCL in dioxane to prepare printing ink, then complete the printing process at temperatures ranging from –25 to –35 °C. After printing, the scaffold is freeze-dried to remove dioxane [95, 108]. As shown in Fig. 2, PLCL porous scaffolds are prepared via the LTD printing technology. The SEM images of the scaffolds show that their surfaces contain a microporous structure with a size range of 6.9–12.1 μm [108]. Unlike the FDM and SLS methods, the LTD printing process is carried out at low temperatures, which preserves the bioactivity of temperature-sensitive bioactive agents, such as growth factors and extracellular matrix proteins [109, 110].

### Supercritical CO_2_ foaming

**Fig. 3:**
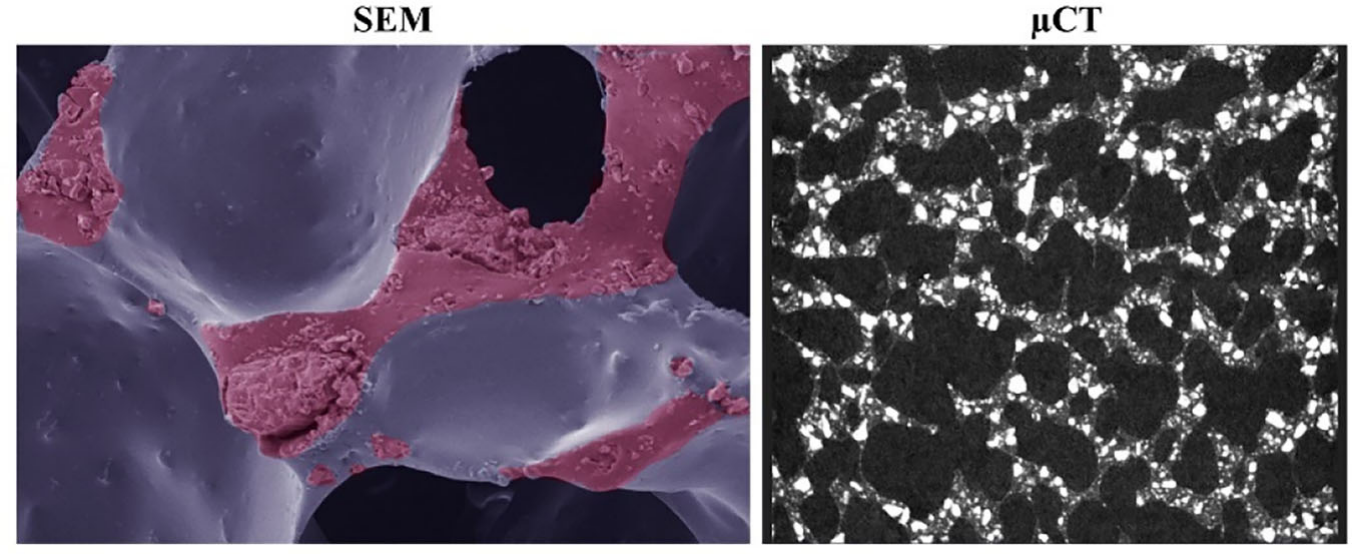
Characterization of scCO₂ porous scaffolds of PLCL/β-TCP Composites using SEM and μCT [13]

ScCO₂ refers to the fluid state of carbon dioxide when it exceeds its critical temperature (31.1 °C) and critical pressure (7.38 MPa). In this state, carbon dioxide has both the solubility of a liquid and the high dispersibility of a gas, and is non-toxic and non-residual [111]. Using this property of scCO₂, porous PLCL scaffolds can be prepared. The fabrication process mainly involves three steps: gas adsorption, depressurization-induced foaming, and curing [112–114]. As shown in Fig. 3 a porous bone repair scaffold was prepared using PLCL/β-tricalcium phosphate (PLCL/β-TCP) as the raw material and scCO₂ foaming technology. The scanning electron microscopy (SEM) images reveal that the purple region represents the smooth internal pore surfaces of the scaffold, while the pink region shows the cross-section, where β-TCP particles are uniformly dispersed. The micro-computed tomography (μCT) results indicate that the scCO₂ technology generated a large number of uniform and interconnected pore structures inside the scaffold [13]. PLCL scaffolds with tailored pore sizes, porosity, and pore size distribution can be fabricated by adjusting pressure, temperature, depressurization rate, and saturation time [115]. ScCO₂ method eliminates the need for organic solvents and involves low processing temperatures [116]. When adding temperature-sensitive modification factors or biologically active factors to the material, this method can prevent the inactivation of bioactive additives during fabrication [117]. In addition, PLCL scaffolds prepared by the supercritical carbon dioxide method contain abundant pore structure, which facilitates cell adhesion and proliferation, interstitial fluid transport, and vascular and tissue ingrowth within the scaffold, so they have many applications in bone tissue engineering, nerve tissue engineering, vascular tissue engineering and cartilage repair [13, 118–120].

### Membranes

**Fig. 4:**
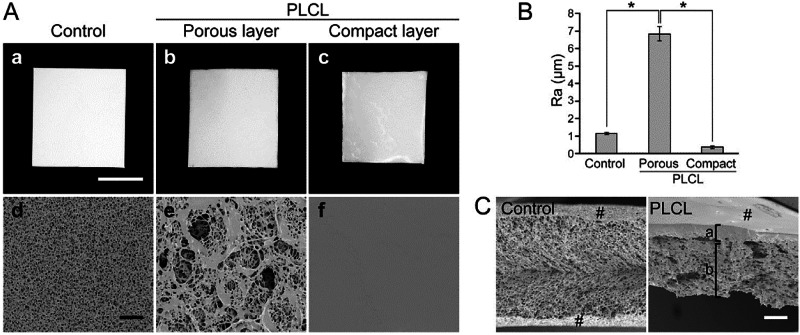
PLCL membrane with the compact layer and porous layer. **A** Macroscopic and microscopic images of the PLCL membrane (scale bar: 100 μm), **B** surface roughness (*n* = 5, *p < 0.05) and **C** microscopic image of the cross-section (scale bar: 100 μm) [123]

Solution casting is the most fundamental method for fabricating PLCL membranes. The PLCL is dissolved in volatile organic solvents such as chloroform and dichloromethane, and then the solution is uniformly cast onto a flat substrate, such as a glass slide or silicone rubber sheet, and after the solvent is volatilized, a film is formed [121]. The films prepared by this method usually have a relatively dense microstructure, so they have good elasticity and flexibility [48]. By simply adjusting the content of PLCL in the solution and the area of the receiving plane, PLCL films of different thicknesses can be obtained relatively easily. In the process of preparation, bioactive modifiers can also be added to give the film different biological properties [122]. As shown in Fig. 4, a kind of bilayer membrane was prepared *via* a two-step freezing and lyophilization process using a PLCL solution. However, this preparation method also has its shortcomings: the organic solvent introduced in the preparation process needs to be fully cleaned and removed; otherwise, residual surface organic solvents may compromise material biosafety [123]. In addition, the mechanical strength of the PLCL of the thin-film structures is weak, and it is unsuitable for load-bearing biomedical applications, such as bone tissue and cartilage tissue.

### Aerogel scaffolds

**Fig. 5:**
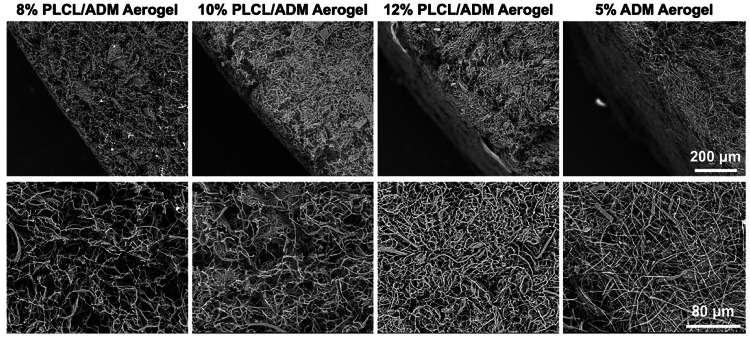
SEM images of PLCL/ADM and ADM aerogels scaffold [126]

Aerogel scaffolds refer to a class of scaffolds with low density, high porosity and specific surface area. The biomimetic structure of aerogel scaffolds mimics ECM architecture, providing a favorable environment for cell growth [124, 125]. There are many methods for preparing aerogel scaffolds [124]. As shown in Fig. 5, nanofibers were fabricated from PLCL and acellular dermal matrix (ADM) *via* electrospinning. Subsequently, the PLCL/ADM nanofibers were cut into short fibers, which were then homogenized and dispersed at high speed in *tert*-butanol with different concentrations. Finally, after freeze-drying, the material eventually forms an aerogel scaffold, which is suitable for the rapid repair of full-thickness skin defects [126]. The interconnected pores and high porosity of aerogel scaffolds provide an ideal environment for cell adhesion and proliferation, and this structure facilitates the exchange of nutrients [127, 128]. The high specific surface area enables efficient loading of therapeutic agents for the treatment of damaged tissues and organs [129, 130]. In different biomedical application fields, different materials and bioactive factors can be selected as the raw materials, and appropriate aerogel scaffold preparation methods can be chosen to meet the needs of tissue engineering repair [131–134]. With the emergence of new technologies for aerogel scaffold preparation, PLCL-based aerogel scaffolds will emerge at the forefront of the tissue engineering repair field.

In biomedical engineering, PLCL offers diverse processing methodologies, including electrospinning, 3D printing, scCO₂ foaming, membrane fabrication, and aerogel scaffold preparation, each with distinct characteristics and tailored application scenarios. Electrospinning enables the fabrication of fibrous materials with a high specific surface area, making them suitable for vascular and nerve repair. 3D printing facilitates the construction of complex, customized structures, where different technical approaches exhibit respective advantages and limitations (e.g., LTD can preserve bioactive factors). ScCO₂ foaming is distinguished by solvent-free residue and low-temperature compatibility, thereby promoting tissue growth. Solution casting for membrane preparation is a straightforward process but requires thorough solvent removal, and the resulting membranes possess inadequate mechanical strength. Aerogel scaffolds, characterized by high porosity and specific surface area, are applicable for skin repair and other analogous applications. Tuning process parameters allows the production of PLCL-based materials with target properties, thus meeting the requirements of diverse tissue engineering scenarios.

## Applications of PLCL in tissue engineering

### Wound healing

The applications of PLCL in wound healing are shown in Table 3. As the largest organ of the human body, skin has the functions of barrier, protection, perception and regulation. If damaged, it can lead to infections, loss of function and even life-threatening conditions. The skin consists of three main layers: epidermis, dermis, and subcutaneous tissue. After skin injury, the body itself goes through three stages of inflammation, proliferation and remodelling to achieve skin wound healing and repair, but this process is affected by the external environment and internal factors of the body [135]. External conditions such as temperature, humidity, oxygen supply, infection and other factors can affect the wound healing process [136]. The internal factors mainly include age, health status, nutritional status and other factors. For example, in diabetic patients, wound healing is significantly delayed due to the inhibition of angiogenesis and dysregulation of inflammation in the hyperglycemic environment [137].

**Table 3:** Applications of PLCL in Wound healing

Aim	Composite material	Processing method	Bioactive factor	Effect	Ref.
Skin wound healing	Collagen	3D printing	Minimal functional unit of skin (MFUS)	Promote skin wound healing in rats	[275]
Skin wound healing	–	Electrospinning	Human fibroblast-derived ECM (hFDM)	Promote cell proliferation and neovascularization	[276]
Infected wound healing	Sr ZnO/PDA	Electrospinning	–	Promotes wound angiogenesis, antibacterial and anti-inflammatory properties	[277]
Diabetic wound healing	–	Electrospinning	Fibrinogen	Promote fibroblast accumulation and collagen production	[278]
Skin wound healing	–	Electrospinning	ADSCs; P123	Promotes microvascular formation and wound healing	[279]
Skin wound healing	Gelatin	Electrospinning	–	Promotes wound contraction and wound healing	[280]
Skin wound healing	–	Electrospinning	Heart Decellularized Extracellular Matrix (hdECM)	Reduces scarring during wound healing	[281]
Skin wound healing	PVP	Electrospinning	Berberine Chloride	Promotes wound healing	[282]
Skin wound healing	SF	Electrospinning	Oregano essential oil	Inhibit the growth of wound bacteria and promote wound healing	[283]
Diabetic wound healing	CS；Cu	Electrospinning	Decellularized Wharton’s jelly matrix (DWJM)	Promote wound angiogenesis and collagen deposition, inhibit bacterial growth	[2]
Skin wound healing	–	Electrospinning	FGF-2; keratin	Enhance adhesion and promote wound healing	[264]
Infected wound healing	–	Electrospinning	Phenol red; epigallocatechin gallate	Wound status monitoring, antibacterial, antioxidant and anti-inflammatory functions	[147]
Skin wound healing	PNIPAAm	Electrospinning	CIF	Promote fibroblast proliferation and inhibit bacterial growth	[284]
Skin wound healing	ZnO; PEO; sodium alginate	Electrospinning	Quadracycline	High moisture retention and long sustained release of antimicrobial agents	[145]
Diabetic wound healing	PCL	Electrospinning	hPL；FGF-2；VEGF	Sustained release of growth factors promotes chronic wound healing	[26]
Skin wound healing	Gelatin	Electrospinning	EGCG	Promotes wound hemostasis and wound healing	[285]
Skin wound healing	Gelatin;collagen	Electrospinning	VEGFA	Promote vascular network formation, promote wound healing	[146]
Skin wound healing	PCL	Electrospinning	Platelet Lysate	Promote the growth of fibroblasts and endothelial cells	[286]
Skin wound healing	SF	Electrospinning	CFBSA	Broad-spectrum antibacterial ability and good biocompatibility	[287]
Diabetic wound healing	SF	Electrospinning	CPL	Inhibit bacterial growth and promote diabetic wound healing	[288]
Skin wound healing	Ag	Electrospinning	GA	Significant bactericidal effect	[289]
Diabetic wound healing	PVA	Electrospinning	LP；VEGF；Amox	Inhibit bacterial growth, promote vascular regeneration and wound healing	[49]

Wound healing is affected by many factors, so the design of wound dressings needs to take into account many factors. Firstly, wound dressings need to ensure good biocompatibility to avoid inducing immune rejection [138]. At the same time, an ideal dressing has appropriate permeability and moisture retention capacity, providing a humid environment for the wound, while allowing gas exchange to prevent the accumulation of local exudate [139]. It has certain antibacterial properties and even a bactericidal effect to prevent wound bacterial infection. Besides, it can provide the growth factors or cytokines for wound healing, and promote the migration, adhesion and proliferation of fibroblasts at the injured area [140]. PLCL has good biocompatibility, does not cause inflammation when applied to a wound site, and has good biosafety. Through electrospinning technology and 3D printing technology, PLCL can be processed into wound dressings with appropriate porosity and mechanical strength, providing support and protection for wound healing.

**Fig. 6:**
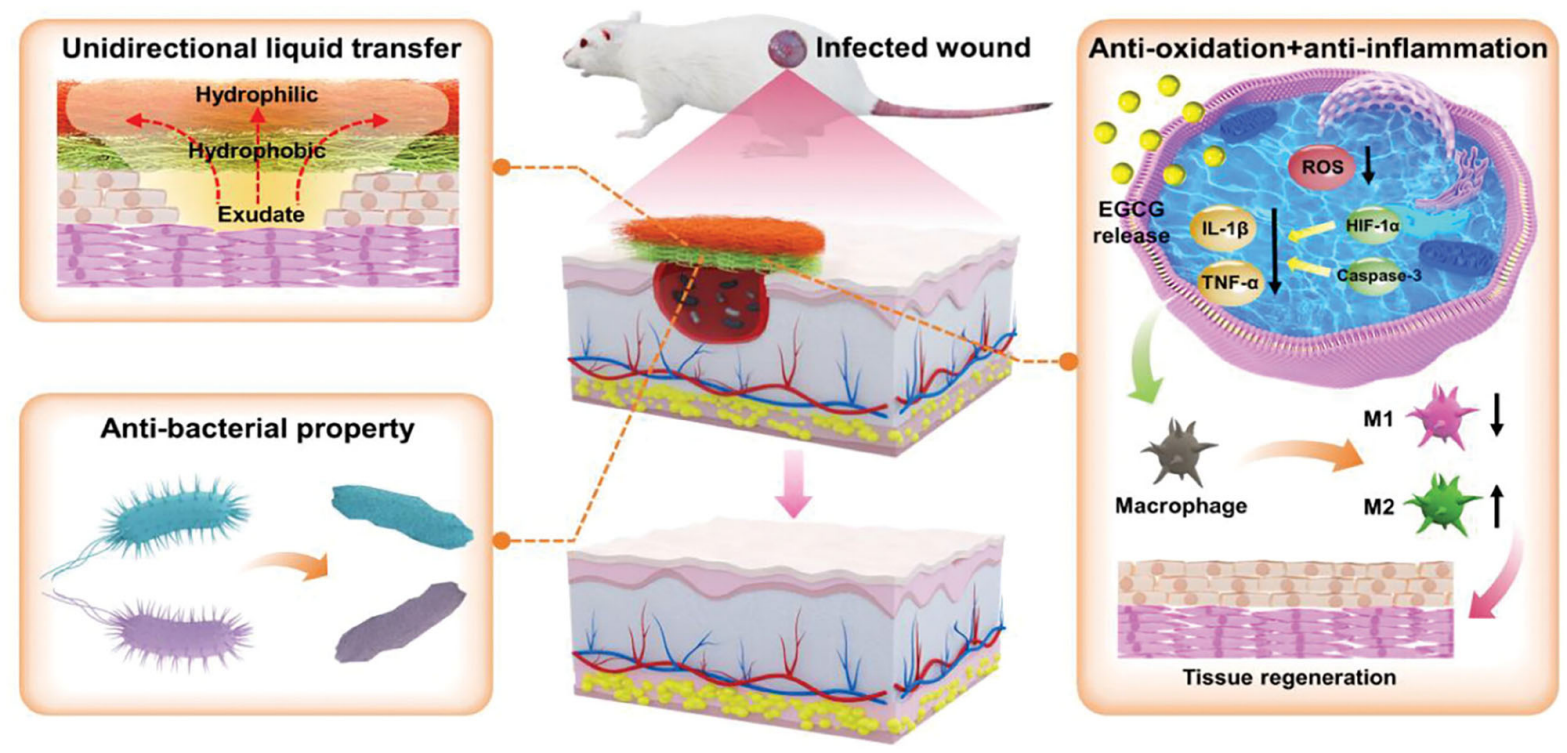
The treatment of infected wound by PLCL Janus dressing [147]

However, PLCL itself is relatively simple and can only provide a mechanical barrier for the wound healing process, so it is necessary to further improve the overall performance of the wound dressing by loading other materials or biological factors [141, 142]. For example, the hydrophilic properties of PLCL materials can be significantly improved by adding collagen, fibroin and chitosan to PLCL. Incorporating electrospun PLCL fibers into hydrogels can significantly enhance the overall mechanical strength of the composite material [143, 144]. Adding antibacterial particles such as silver ions, ZnO or bioactive antibacterial substances such as antibiotics to PLCL can significantly improve the overall antibacterial performance of the material, and at the same time, with the gradual degradation of PLCL, the long-lasting release of antibacterial substances can be achieved, thereby reducing the risk of infection during the entire healing process [145]. Furthermore, growth factors such as FGF and VEGF can be incorporated into or onto the PLCL matrix during fabrication. This promotes the migration, adhesion, and proliferation of fibroblasts, as well as vascular regeneration in the wound area, thereby accelerating the healing process [146]. Deng et al. manufactured a highly stable and multifunctional Janus dressing with unidirectional exudate transfer ability based on PLCL (Fig. 6). The PLCL electrospun fibers were transformed from hydrophobic to hydrophilic through an acid hydrolysis reaction, thus endowing the material with unidirectional liquid transfer ability and high structural stability. On this basis, the addition of phenol red and epigallocatechin gallate further provided wound status monitoring and antibacterial, antioxidant and anti-inflammatory properties for the dressing. The application of Janus dressings to infected wound models promotes wound epithelialization and collagen deposition, reduces inflammatory response, and promotes angiogenesis [147].

PLCL exhibits excellent biocompatibility and high biosafety, and can be fabricated into wound dressings suitable for wound healing via techniques including electrospinning and 3D printing. However, it has a singular function, necessitating the incorporation of materials or bioactive factors (e.g., collagen, antibacterial particles, and FGF) to optimize properties such as hydrophilicity, mechanical performance, and antibacterial activity. Existing studies have developed multifunctional formulations, such as Janus dressings, which effectively promote wound healing. As a biodegradable material for preparing wound dressings, PLCL still has certain limitations. For example, although the degradation rate of PLCL is significantly higher than that of PCL and PLA, it remains relatively slow [148]. Therefore, PLCL is only suitable for the preparation of chronic wound dressings and not suitable for acute wound dressings.

### Vascular tissue engineering

The applications of PLCL in vascular tissue engineering are presented in Table 4. Cardiovascular disease is one of the leading causes of death worldwide, and vascular graft transplantation and repair technology are key means to treat many vascular-related diseases. However, due to the limitations of autologous vascular donors and the biocompatibility of artificial materials, the development of ideal vascular substitutes has become an important goal of vascular tissue engineering. Although the currently used synthetic vascular grafts such as PTFE and PET have good mechanical properties, they lack endothelialization ability in small-caliber vessels (diameter <6 mm) and are prone to thrombosis, so the design of tissue engineering materials for artificial vascular grafts still needs to be further improved.

**Table 4:** Applications of PLCL in Vascular tissue engineering

Aim	Composite material	Processing method	Bioactive factor	Effect	Ref.
Small-Diameter Vascular Scaffolds	–	Electrospinning	EPCs-TPS; heparin	Reduce platelet adhesion and promote EPCs adhesion	[24]
Small-Diameter Vascular Scaffolds	PCL; TSF	Electrospinning	–	Good elastic recovery and bursting pressure, good hydrophilicity and protein adsorption properties	[290]
Small-Diameter Vascular Scaffolds	PCL	Supercritical CO2 foaming	–	Improved flexibility and recoverability of scaffolds for better HUVEC growth	[120]
Venous vascular patch	PGA	Electrospinning	BMSCs	Promote the regeneration of endothelial cells and vascular smooth muscle in the patch area	[291]
Vascular graft	Collagen; SF	Electrospinning	–	Good tensile mechanical strength and elongation promote HUVECs aggregation and collagen production	[292]
Vascular graft	Collagen; Gelatin	Electrospinning	–	Smooth inner wall structure, good hydrophilicity and mechanical properties	[293]
Vascular graft	–	Electrospinning	spidroins	Improve the tensile strength and elongation of vascular scaffolds, reduce the fiber diameter, increase the porosity and hydrophilicity of scaffolds, and improve the cell adhesion and proliferation activity of the materials	[294]
Small-Diameter Vascular Scaffolds	TM NPs	Electrospinning	–	Improve the hydrophilic and anticoagulant properties, better tensile strength and elongation than coronary artery	[295]
Small-Diameter Vascular Scaffolds	PEG-CS	Electrospinning	–	Good endothelial cell compatibility and blood compatibility	[296]
Vascular graft	–	Electrospinning	elastin	Anti-thrombotic, reduce platelet adhesion, improve blood compatibility	[297]
Vascular graft	–	Electrospinning	TSF	Increase the radial tensile strength (2x) and axial Young modulus (3x)	[298]
Vascular graft	PLGA-PPO-PLGA	Electrospinning	fibrinogen	Promote the growth of smooth muscle cells on the scaffold surface	[299]
Small-Diameter Vascular Scaffolds	SF	Electrospinning	Heparin	Promote endothelial cell growth and reduce platelet adhesion	[156]
Vascular graft	–	Ink printing, wet spinning, and electrospinning	–	Enhance the formation of endothelial cells on the surface of the scaffold monolayer, promote the growth of vascular smooth muscle cells and fibroblasts and establish connections	[154]
Vascular graft	PLLA; PCL	3D printing		Good bending fatigue and compression resistance, suitable degradation time	[300]
Vascular graft	PGA	Electrospinning	BMSCs	Good vascular compatibility promotes the expression of eNOS in the stent	[301]
Vascular graft	PLA	Electrospinning	–	Promote the formation of vascular intima and prevent calcification deposits	[302]
Small-Diameter Vascular Scaffolds	Collagen; CS	Electrospinning	Heparin	Suitable mechanical strength and flexibility, good burst pressure, continuous release of heparin, good anti-platelet adhesion ability	[158]
Small-Diameter Vascular Scaffolds	PU	Electrospinning	–	Mechanical properties similar to natural vascular grafts, better vascular patency rate	[303]
Vascular graft	PCL	Electrospinning	SP; heparin	Improve stent vascular compatibility, recruit mesenchymal stem cells and promote endothelialization	[157]
Vascular graft	–	Freeze-drying; melt-spinning	–	Better tensile strength and bursting pressure, good swelling rate and porosity	[304]
Small-Diameter Vascular Scaffolds	–	Electrospinning	Heparin; silk-gel	Inhibit thrombosis and maintain long-term vascular patency rate	[305]
Small-Diameter Vascular Scaffolds	GelMA	Electrospinning	–	The bursting pressure and tensile strength of the scaffold were increased to promote the adhesion and growth of endothelial cells	[306]
Small-Diameter Vascular Scaffolds	PU	Electrospinning	Heparin	Better patency rate, adequate mechanical properties and remodeling ability	[307]
Vascular graft	Collagen	Electrospinning	Heparin; VEGF; PDGF	Faster rate of endothelialization and smooth muscle infiltration	[27]
Vascular graft	–	3D printing	–	A multi-branch vascular stent array with high precision was constructed	[155]
Small-Diameter Vascular Scaffolds	–	Electrospinning	hCOLIII; PC	Good cell compatibility, blood compatibility and anti-calcification performance, inhibit endometrial hyperplasia	[266]
Small-Diameter Vascular Scaffolds	–	Electrospinning	rhCOLIII	Reduce platelet adhesion and promote the adhesion and growth of endothelial cells in the scaffold	[308]
Vascular graft	PDO	Electrospinning	–	Highly simulates the “J-shaped” stress-strain curve of natural vascular grafts, with good biocompatibility	[309]
Vascular graft	CS	Electrospinning	–	Good mechanical properties, better anticoagulation properties and anti-hemolysis properties	[310]
Vascular graft	PLLA; PCL	Electrospinning	–	Matched functional growth of endothelial cells and smooth muscle cells	[311]
Vascular graft	PEG; PLLA	Membrane		Good adhesion of endothelial cells	[312]
Small-Diameter Vascular Scaffolds	–	Electrospinning	Lys;	Anti-thrombotic and anti-inflammatory effects, promotes cell-fiber and cell-cell interactions, accelerates the formation of endothelial monolayer	[313]
Small-Diameter Vascular Scaffolds	Cu, HA	Electrospinning	keratin	Promote the regeneration and migration of endothelial cells, inhibit the excessive proliferation of endothelial cells, prevent vascular calcification, reduce inflammation	[314]
Valve scaffold	PLLA	3D printing	–	Good mechanical properties, excellent biocompatibility and vascular formation in vivo	[315]
Small-Diameter Vascular Scaffolds	PLGA; PLLA;	Thermally induced phase separation	SDF-1; heparin	Stronger anticoagulant effect, promote EPC recruitment and endothelial cell migration and proliferation, accelerate vascular stent endothelialization, inhibit smooth muscle cell proliferation	[316]
Vascular graft	PEO	Electrospinning	tanshinone	Better anti-platelet adhesion effect, promote cell proliferation and directed growth	[317]
Small-Diameter Vascular Scaffolds	–	Electrospinning	VEGF; heparin	Long-term continuous release of heparin, good anticoagulant performance; Promote the proliferation and endothelialization of endothelial cells on the scaffold surface	[318]
Small-Diameter Vascular Scaffolds	SF	Electrospinning	–	Better flexibility and burst pressure than biological vascular grafts, good biocompatibility	[319]

**Fig. 7:**
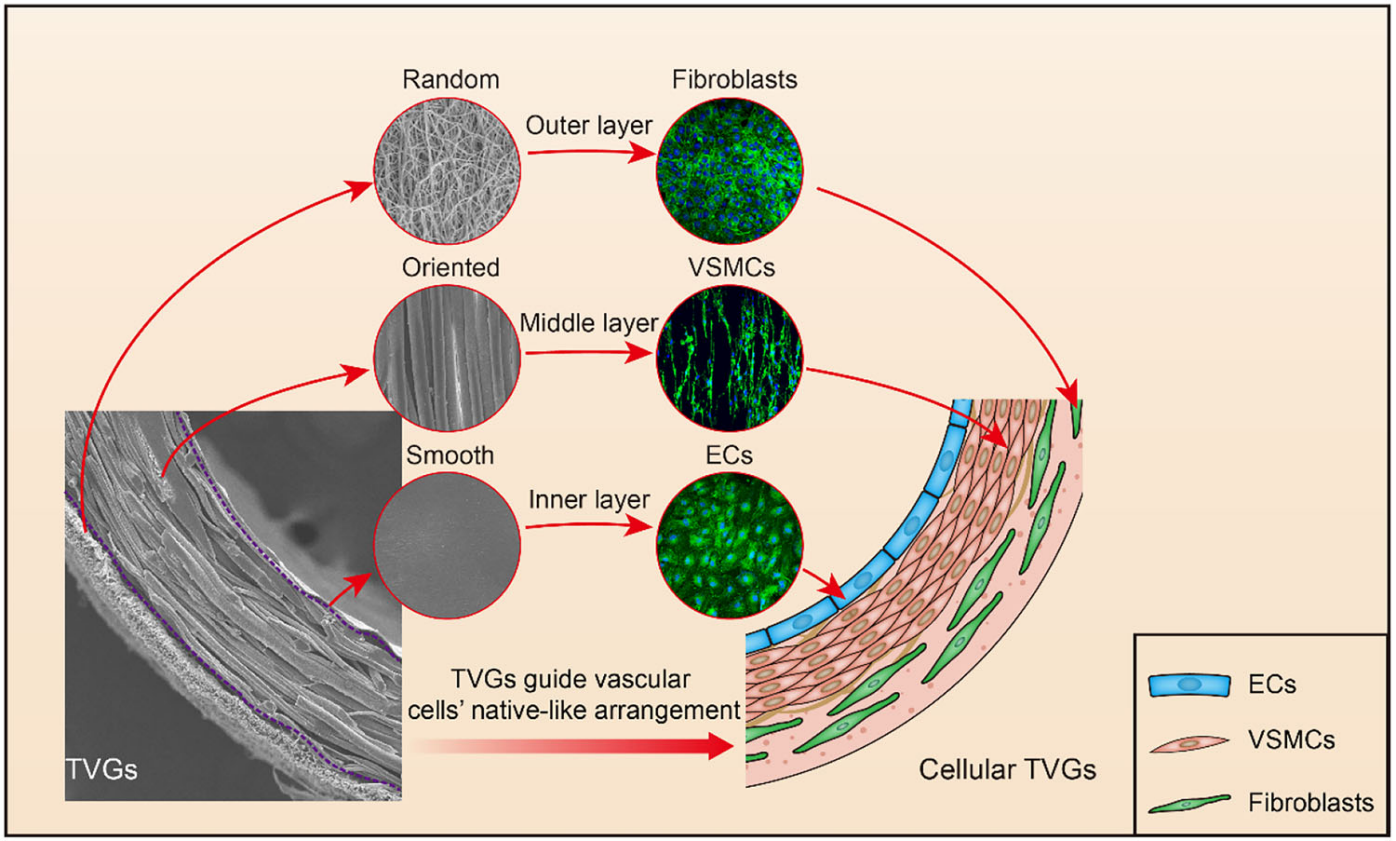
Triple-layer structure of PLCL vascular graft [154]

An ideal vascular tissue engineering material should have the following characteristics: first, it should have good biocompatibility, and on this basis, it should have suitable mechanical properties. Vascular grafts need to withstand the impact of blood flow and the extrusion and traction from surrounding tissues in the physiological state, so they need to have certain mechanical properties such as tensile properties and elongation at break, and adequate anti-fracture strength [149]. In addition, the vascular stent should have good anti-platelet aggregation ability and anti-thrombosis ability, which is required to be long-term and continuous [150]. At the same time, the inner layer of the scaffold should have good biological activity, which can promote the migration, aggregation, adhesion and growth of vascular endothelial cells, and accelerate the process of vascular endothelialization [151]. Finally, the vascular scaffold should have a proper degradation time, so that it can be synchronized with the formation of new tissue [152]. Electrospinning technology can make PLCL into vascular scaffolds with fiber structure, which provides an ideal environment for cell adhesion and migration. On this basis, directional spinning can be further prepared, or composite vascular scaffolds with double or triple structure can be prepared by different technical methods, so as to better simulate vascular structure, thus providing better mechanical properties [153]. Yuan et al. used ink printing, wet spinning, and electrospinning techniques to sequentially prepare a three-layer PLCL vascular graft tri-layer vascular graft (TVG) consisting of a smooth inner layer, a circumferential fiber middle layer, and an outer layer with randomly distributed fibers (Fig. 7). TVGs have kinking resistance and adequate mechanical properties (tensile strength, elastic modulus, suture retention strength, and burst pressure) and are equivalent to the clinical gold standard grafts in clinical applications, namely human saphenous vein and human internal mammary artery. The layered structure of TVGs shows obvious guiding effects on specific vascular cells, including enhancing endothelial cell (EC) monolayer formation, facilitating vascular smooth muscle cell (VSMCs) alignment and elongation, and promoting fibroblast proliferation and junction establishment. 3D printing technology can realize the customized design of artificial vascular grafts and the design and preparation of complex vascular structures [154]. Using PLCL as the model material, Han et al. fabricated a multi-branch vascular scaffold array with high accuracy through 3D printing technology based on material plastination process. This scaffold exhibits high structural fidelity and good mechanical properties, which can meet the construction of complex organ model structure in clinical applications [155].

In addition to the optimization of mechanical properties of PLCL artificial vascular scaffolds, the design of its internal surface is often contradictory when applied in vivo. On the one hand, a smooth inner layer structure can reduce the aggregation of platelets and reduce the probability of thrombosis; on the other hand, smooth surface is not conducive to the growth of vascular endothelial cells and endothelial reconstruction of vascular grafts. In order to solve this problem, most studies choose to add heparin to PLCL vascular stents to enhance the anticoagulant performance of the stents [156–158]. In addition, PLCL vascular stent also needs to play a regulatory role in the process of vascular regeneration and repair, which requires the joint action of vascular endothelial cells and smooth muscle cells. Endothelial cells can reconstruct the intima structure of vessels and regulate vascular regeneration by secreting growth factors (VEGF) and active factor (NO) [159]. Smooth muscle cells give elasticity and contractility to vascular grafts in the media layer of vascular grafts, while secreting extracellular matrix to stabilize vascular structure [160]. Adding growth factors such as VEGF and PDGF to PLCL vascular scaffolds can effectively promote vascular endothelialization, accelerate migration and aggregation of endothelial cells and smooth muscle cells, and promote vascular regeneration and reconstruction [161]. However, it should be noted that the excessive proliferation of endothelial cells and smooth muscle cells will cause intimal hyperplasia, lumen narrowing, and local poor blood flow, which will lead to the formation of thrombosis and calcified plaque [162]. Therefore, this potential issue warrants careful consideration in the design of PLCL-based vascular biomaterials.

Vascular scaffolds can be fabricated from PLCL *via* techniques including electrospinning and 3D printing. These scaffolds are capable of mimicking native vascular architectures, exhibit favorable mechanical properties, and thereby meet clinical requirements. An ideal vascular material must satisfy multiple criteria, such as excellent biocompatibility and antithrombotic activity. The anticoagulant performance of PLCL scaffolds can be enhanced by heparin incorporation, while loading with growth factors (e.g., VEGF) effectively promotes vascular endothelialization and regeneration. However, the inner surface design of PLCL scaffolds presents inherent contradictions, and caution should be exercised regarding potential issues (e.g., intimal hyperplasia) induced by excessive cellular proliferation.

### Cardiovascular tissue engineering

The application of PLCL materials in myocardial tissue engineering mainly focuses on two aspects. On one hand, PLCL-based cardiac patches are used in surgical interventions and for repair following myocardial injury. On the other hand, the artificial heart valve designed and prepared with PLCL as the matrix material is used for artificial valve replacement after valve resection in heart valve disease. The application strategies and research focus for PLCL in these two scenarios differ significantly. The heart patch needs to have dynamic mechanical properties, similar to the elastic modulus and flexibility of myocardial tissue, in order to withstand the mechanical stress caused by the beating of the heart. Furthermore, the patch should have long-term mechanical stability and provide sufficient mechanical strength and integrity for an extended period after implantation, until the formation of new tissue [163]. On this basis, the patch should also have certain biological functions, which can simulate the microstructure and biological function of natural heart tissue, and provide a suitable extracellular matrix environment [164]. At the same time, it can be used as a carrier to slowly release growth factors such as VEGF and bFGF to promote angiogenesis and tissue regeneration [165]. The mechanical properties of PLCL can meet the requirements of the heart patch, and the biological properties of the patch can be further improved by loading growth factors or cells onto the PLCL surface [166]. Sugiura et al. prepared a biodegradable patch using PGA and PLCL as materials, seeded with cardiomyocytes derived from human induced pluripotent stem cells onto the patch, which was then implanted over the myocardial defect. The results showed that the PGA-PLCL patch loaded with cardiomyocytes could promote the healing of the myocardium defect area and express more α-actin [167].

**Fig. 8:**
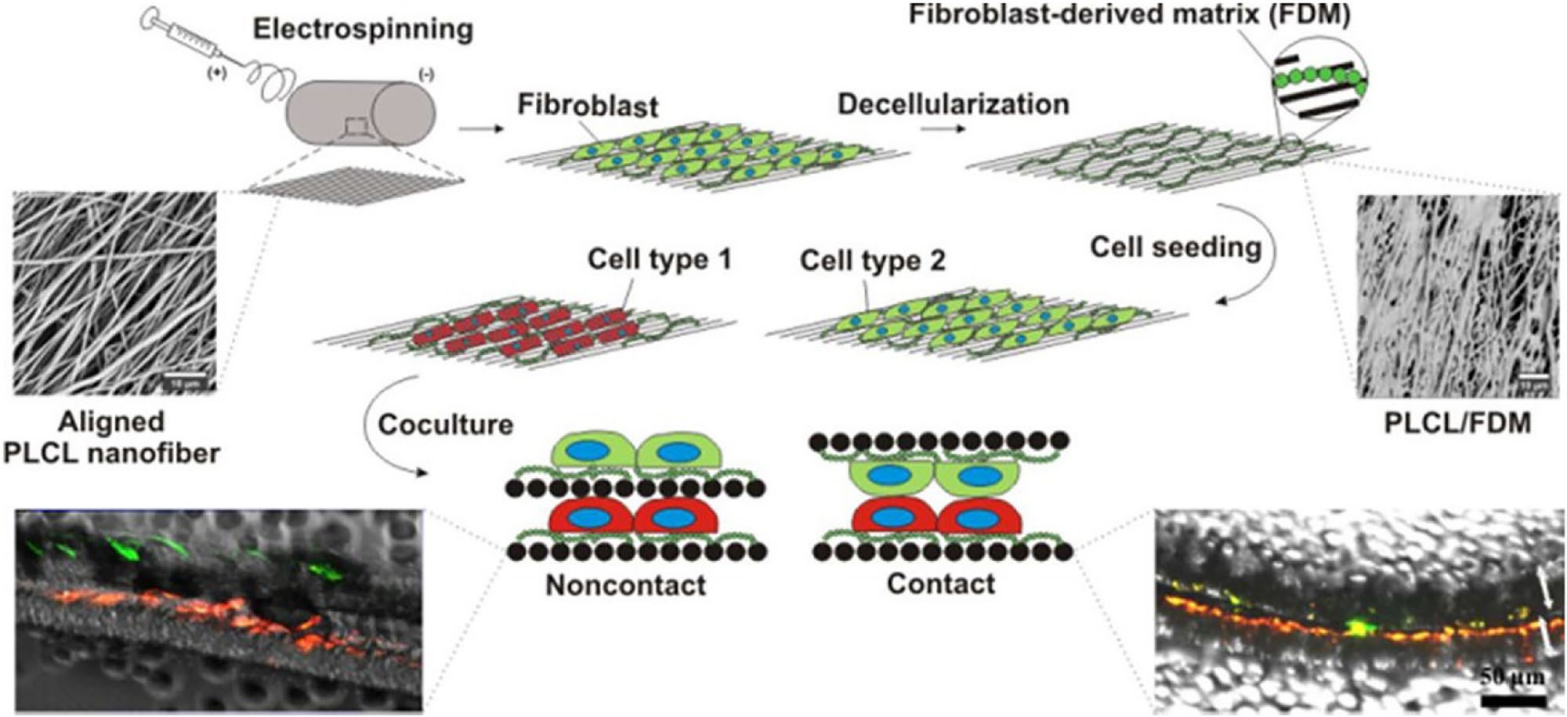
The process of fabricating PLCL/FDM novel platform for cardiomyocyte culture and coculture [173]

The design of heart valves has higher material requirements. Heart valves must continuously open and close while enduring constant hemodynamic stresses. Moreover, the direction of valve movement and the pressure distribution vary with their anatomical location. Consequently, the matrix materials used for heart valve design demand superior and anisotropic mechanical properties, presenting a significant challenge for PLCL alone [168, 169]. In addition, the valve surface needs to withstand the continuous impact of blood flow, making it prone to deposits such as calcification and thrombosis. Therefore, imparting anti-thrombogenic and anti-calcification properties is crucial for artificial heart valves, capabilities that PLCL inherently lacks, and PLCL itself does not have these capabilities [170]. To solve this problem, the researchers formed the new composite material by incorporating different proportions of other materials into the PLCL matrix to improve the mechanical strength of the material so that it can meet the needs of the heart valve [149, 171]. Using PCL and PLCL as matrix materials, Snyder et al. prepared an elastic three-layer PCL/PLCL lobular substrate with natural tensile, bending and anisotropic properties through electrospinning technology. This composite material has better anisotropy and flexibility, and can better meet the needs of heart valves [172]. Suhaeri et al. constructed a stroma-coupled and aligned electrospun fiber derived from fibroblasts (Fig. 8). This novel platform can provide a good growth environment for cardiomyocytes, and neonatal rat cardiomyocytes grown on the new platform have better maturity and cell viability [173]. Wang et al. constructed a composite electrospinning biological scaffold composed of fibroin protein and PLCL, and in vitro experiments showed that the PLCL/SF scaffold had good biocompatibility and anti-calcification ability [174]. Wang et al. used PLCL, gelatin, hyaluronic acid, and fibroin proteins to construct a bionic three-layer tissue-engineered heart valve to mimic the fiber, sponge, and ventricular layers of a natural heart valve. The results of subcutaneous implantation in rats showed that the biomimetic composite scaffold had appropriate mechanical strength, low calcification deposition and good regenerative ability [175].

PLCL is primarily utilized in myocardial tissue engineering for the fabrication of cardiac patches and artificial heart valves. Cardiac patches require matching the dynamic mechanical properties and long-term stability of myocardial tissue, a requirement that PLCL can meet. Incorporating growth factors or cells into PLCL enhances its biological functionalities, and it has been demonstrated to promote the healing of myocardial defects. Artificial heart valves impose more stringent material demands; PLCL must be combined with other materials to improve mechanical performance, antithrombotic activity, and anti-calcification properties, with relevant composite scaffold studies having exhibited application potential. Nevertheless, most current PLCL-based myocardial tissue engineering scaffolds remain in the in vitro research stage, and their specific in vivo applications and efficacy await further verification.

### Nerve tissue engineering

The applications of PLCL in nerve tissue engineering are presented in Table 5. Nerve tissue engineering represents a vital strategy for repairing and regenerating damaged neural tissues. It involves designing biomimetic materials and scaffolds to provide a conducive growth environment for nerve cells, thereby promoting neural repair. Due to the existence of environmental inhibitory factors in the central nervous system, the ability of nerve axon regeneration is limited, and often cannot get a good therapeutic effect [176, 177]. In contrast, the peripheral nervous system (PNS) has a relatively simpler structure. Injury to the PNS typically does not directly damage the neuronal cell body but rather involves axonal breakage and myelin destruction [178]. Consequently, the PNS exhibits greater reparative capacity and a more favorable prognosis, which also provides a promising application scenario for tissue engineering materials [178]. For peripheral nerve regeneration, these scaffolds must first provide a suitable external environment for nerve fiber growth, using biomaterials to bridge the gap between interrupted nerve fibers. They should also possess sufficient mechanical strength to protect the damaged nerve tissue from secondary compression and invasion by surrounding tissues [179]. The directional microstructure was further designed to promote the directional growth of nerve fibers [180]. In addition, the scaffold should also provide a suitable local microenvironment for nerve growth, such as promoting axon growth by carrying neurotrophic factors such as NGF and loading substances such as MgO to enhance anti-inflammatory ability and reduce local inflammatory response [181, 182].

**Table 5:** Applications of PLCL in nerve tissue engineering

Aim	Composite material	Processing method	Bioactive factor	Effect	Ref.
Nerve tissue engineering	MWCNT	Electrospinning	–	Promote DRG neurite growth	[320]
Sciatic nerve defects repair	SF; PPy	Electrospinning	NGF; TA	The proliferation and migration of PC12 cells under oxidative stress were enhanced and apoptosis was reduced	[321]
Nerve tissue engineering	Collagen	Electrospinning	MSCs	Promote the differentiation of MSCs into neuro phenotypes	[184]
Sciatic nerve defects repair	–	Electrospinning	ECM	Improve the mechanical strength of nerve conduit, promote cell adhesion and proliferation	[322]
Sciatic nerve defects repair	Collagen	Electrospinning	–	Promotes regeneration of axons and myelin	[183]
Sciatic nerve defects repair	Gelatin	Electrospinning	–	Induces axon regeneration, promotes myelin repair and functional recovery	[270]
Nerve tissue engineering	–	Electrospinning	laminin	Promote the proliferation of Schwann cells on the surface of the material, and express neural-related marker proteins	[323]
Sciatic nerve defects repair	–	Membrane	methylcobalamin	Promote Schwann cell proliferation and elongation, induce sciatic nerve defect regeneration	[324]
Nerve tissue engineering	PLGA; PPy	Electrospinning; 3D printing	–	Better conductivity and hydrophilicity, good biocompatibility	[271]
Sciatic nerve defects repair	PPy; SF	Electrospinning	-	Promote myelination after nerve injury	[188]
Sciatic nerve defects repair	–	Electrospinning	decellularized epineurium (DEP)	Good biocompatibility and mechanical properties promote the repair of nerve defects	[265]
Nerve tissue engineering	PPy; SF	Electrospinning	Electrical stimulation	Improve the hydrophilicity and conductivity of the material, promote PC12 cell differentiation and axon extension	[325]
Sciatic nerve defects repair	–	Electrospinning	NGF; KHI peptide	Promote the adhesion and axon elongation of blood flourishing cells	[185]
Nerve tissue engineering	PAni	Electrospinning	–	Promote PC12 cell differentiation and neurite growth	[326]
Nerve tissue engineering	PDA; GN	Electrospinning	–	Promotes myelin growth and nerve regeneration	[327]
Sciatic nerve defects repair	PVDF; PEDOT	Electrospinning	–	Regulating the immune microenvironment promotes Schwann cell recruitment and myelination, and promotes nerve regeneration	[187]
Sciatic nerve defects repair	SF	Electrospinning; Freeze-drying	–	Enhance the proliferation of SCs and promote the recovery of nerve function	[328]
Nerve tissue engineering	–	Electrospinning	norepinephrine	Promote directional growth of nerve fibers and promote neurite extension	[329]
Nerve tissue engineering	PLGA	Electrospinning; 3D printing	–	Improve the surface roughness and hydrophilicity of the scaffold, better mechanical strength, and promote cell adhesion and proliferation	[330]
Nerve tissue engineering	GO	near-field electrostatic printing	Electrical stimulation	Improve the conductivity of the scaffold and induce the formation of neural networks along the fibers under electrical stimulation	[331]
Nerve tissue engineering	GO	Membrane	–	Regulates phenotypic transformation of macrophages, promotes nerve regeneration	[332]
Sciatic nerve defects repair	SF; PEDOT	Electrospinning; Freeze-drying	–	Good single point performance promotes proliferation, maturation and myelination of SCs	[186]
Nerve tissue engineering	PANi; NF	Electrospinning	NGF	Promote PC12 directional growth, increase the number of neurites	[333]

**Fig. 9:**
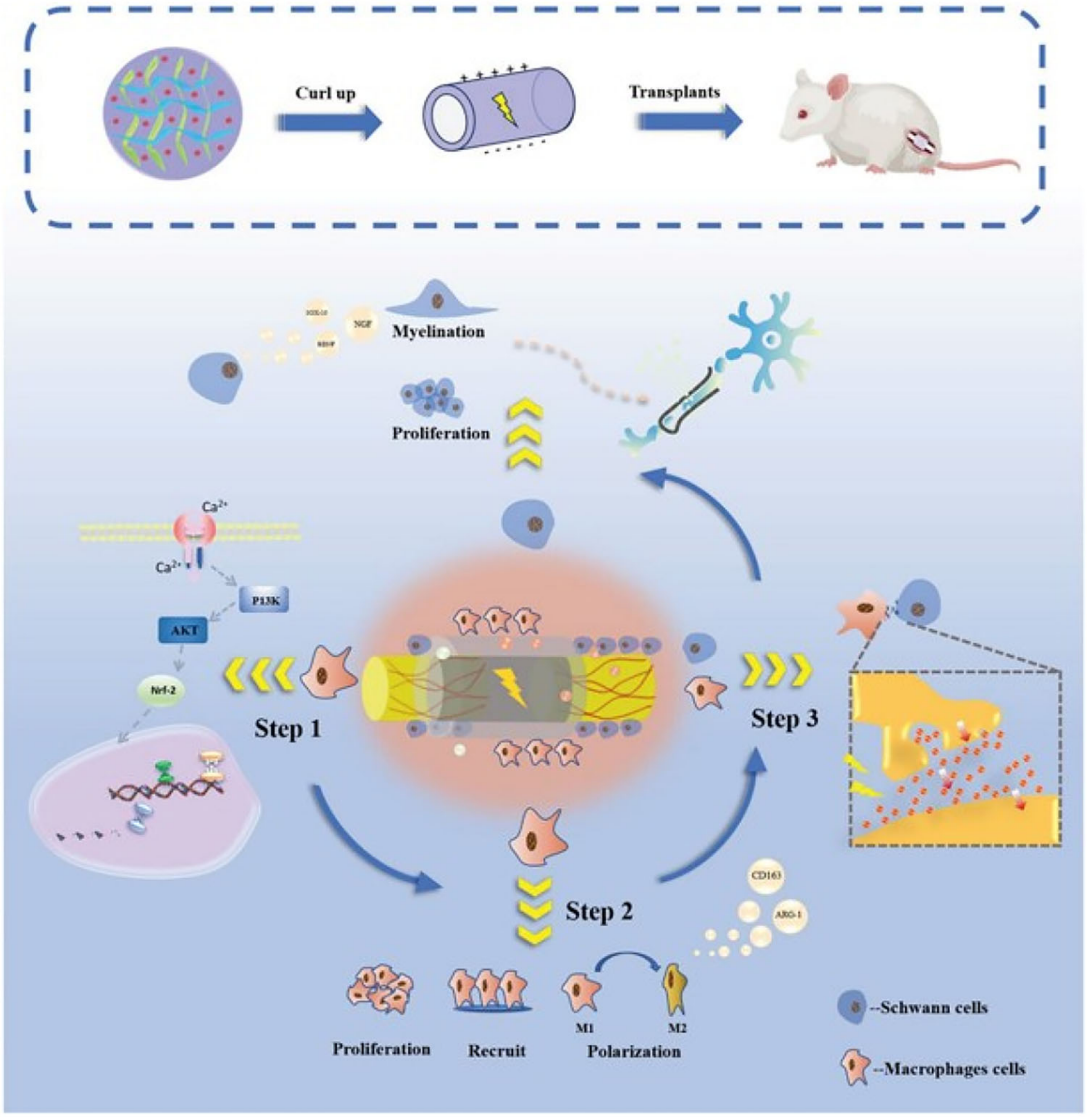
Schematic diagram of PVDF/PLCL/PEDOT electroactive artificial nerve conduit indirectly promoting the repair of damaged peripheral nerves by modulating the immune microenvironment [187]

PLCL has appropriate mechanical strength and degradation rate, which can meet the mechanical strength requirements of scaffolds or catheters during nerve regeneration and healing. Directional spinning and patterned electrospinning can also be prepared by electrospinning technology, so as to promote the directional growth of axons. By introducing collagen, gelatin and fibroin into PLCL, the cell adhesion ability of PLCL nerve conduit can be significantly improved, and the migration adhesion and growth of nerve cells can be promoted [183, 184]. On this basis, bioactive ingredients such as NGF can be further added to the nerve conduit, which can further enhance the induction and promotion effect of the scaffold on nerve fiber growth [185]. In addition, nerve tissue itself has electrical activity, and exogenous electrical stimulation can improve the elongation rate of nerve fibers and speed up the repair process. Although PLCL itself is non-conductive, its electrical properties can be enhanced by incorporating electroactive components such as PVDF, PEDOT, GO, or PPy (Fig. 9) [186, 187]. This not only improves the conductivity of PLCL neural conduits but can also maintain good biocompatibility. It can be used in combination with exogenous electrical stimulation to further promote the regeneration and repair of nerve tissue. Sun et al. wrapped PPy monomer into PLCL/SF electrospun nanofibers by in-situ oxidative polymerization, which significantly improved the electrical conductivity of nerve ducts while ensuring good biocompatibility, and promoted the proliferation and differentiation of PC12 cells and Schwann cells and axon extension on PPy coated nanofibers by applying exogenous electrical stimulation [188]. Wang et al. constructed a PVDF/PLCL/PEDOT neural catheter with both conductive and piezoelectric electroactivity. This electroactive material can regulate the immune microenvironment by activating the PI3K/AKT-Nrf2 signaling pathway, thereby promoting the polarization of macrophages towards M2 anti-inflammatory phenotype, and promoting Schwann cell recruitment and myelination [187].

Despite its broad applicability in neural tissue engineering, several challenges must be addressed when utilizing PLCL. First, the degradation rate of PLCL must be carefully matched to the regeneration rate of peripheral nerves. Excessively rapid degradation will lead to the loss of mechanical support of scaffolds, and the acidic products generated by the degradation of PLCL will also adversely affect nerve growth. Too long degradation time may also interfere with the growth of new nerve tissue [179]. Second, PLCL inherently lacks bioactivity, necessitating the incorporation of various biological components (e.g., neurotrophic factors, conductive polymers) to achieve desired functionalities. However, neural repair is a complex, multifactor-regulated process. Incorporating multiple components often requires complex fabrication processes, which can potentially compromise the material’s overall properties and stability. Therefore, future research should focus on developing more integrated bioactive formulations or simpler, more robust fabrication methods to advance PLCL-based neural repair strategies.

### Cartilage tissue engineering

The applications of PLCL in cartilage tissue engineering are presented in Table 6. Cartilage tissue engineering aims to repair and regenerate defects resulting from injury or diseases like osteoarthritis. The versatile nature of PLCL makes it a promising candidate material for cartilage scaffolds. The structure of cartilage is composed of cells and extracellular matrix. Chondrocytes, the sole cell type in mature cartilage, secrete ECM components such as collagen, proteoglycans, and glycosaminoglycans, which maintain the tissue’s structure and function [189]. The extracellular matrix is mainly composed of type II collagen, which provides buffer for cartilage tissue and disperses mechanical load. Cartilage tissue, especially articular cartilage, has a typical layered structure, with the surface layer, middle layer, deep layer and calcification layer successively from the outside to the inside [190]. With the deepening of ECM, collagen components gradually decrease, and ossification and mineralization components gradually increase [191]. Natural cartilage tissue has excellent elastic and mechanical properties, which can produce elastic deformation to provide buffer when stressed, and can be restored to the original form when external force is removed, which requires biological tissue engineering materials to have the same excellent elastic and mechanical properties.

**Table 6:** Applications of PLCL in Cartilage tissue engineering

Composite material	Processing method	Bioactive factor	Effect	Ref.
Collagen	Electrospinning	–	Promote the growth of cartilage cells on the surface of the material	[334]
PLLA	3D printing	–	Similar pressure relaxation time and mechanical strength to the cartilage tissue; promote chondrocyte proliferation and chondrogenesis	[335]
CS	Freeze-drying	–	Promote the uniform distribution of MSC on the surface of the material, promote cartilage formation, and significantly enhance the Young’s modulus of cartilage formation	[336]
–	Gel-pressing method	–	Good mechanical and elastic properties promote the growth of cartilage cells on the surface of the scaffold	[337]
FG; HA	Gel-pressing method	–	Highly elastic scaffold, highly simulated natural cartilage microenvironment, promote cartilage tissue growth	[338]
–	Supercritical CO_2_ foaming	TGF-β3	The long-term and stable release of TGF, enhance the extracellular matrix deposition and cartilage formation of scaffolds	[272]
CS	Freeze-drying		Similar viscoelastic and compressive deformation recovery rates to bovine cartilage, promote cell adhesion and proliferation	[339]
–	Salt impregnation and leaching method	BMSCs	Good mechanical support strength, promote the adhesion growth of cartilage cells and collagen synthesis	[228]
Collagen	3D printing	–	Good biocompatibility and hydrophilicity, better chondrocyte growth morphology	[108]
PHBV	Emulsion solvent evaporation method	–	Higher mechanical strength and compression modulus, more collagen and cartilaginous tissue formation	[340]
HA; SF	Electrospinning	–	Better chondrocyte proliferation and phenotypic maintenance, better mechanical properties	[341]
Gelatin	Electrospinning	Heparin	Higher chondrogenic gene expression and GAGs production	[342]
PLGA	Gel-pressing method	TGF-β1; Pluronic F-127; hASCs	Induce hASCs chondrogenic differentiation and promote chondrogenic integration	[197]
Gelatin	Electrospinning	curcumin; KGN	Maintain the cartilage form of regenerated cartilage, inhibit cartilage ossification, promote cartilage maturity and stability	[17]
PNIPAM; MA; CS	Electrospinning	–	Dynamically regulates the behavior of chondrocytes and promotes the formation of cartilage tissue in vivo	[267]
BG	Electrospinning	chondroitin sulfate	An artificial osteochondral interface with hardness gradient properties	[343]
PLGA；BG	Electrospinning	TGF; BMSCs；chondroitin sulfate	Promote the regeneration of hyaline cartilage and promote the reconstruction and integration of osteochondral interface	[196]
Sulfate alginate	3D printing	TGF	Enhance chondrogenic differentiation and chondrogenesis of MSCs; Promotes collagen deposition	[344]

The mechanical properties of PLCL, particularly its elastic modulus, can be tuned via the lactic acid-to-caprolactone ratio to match that of natural cartilage, meeting the functional demands for repair. Furthermore, by incorporating components like BG and chondroitin sulfate, composite PLCL scaffolds with gradient hardness can be designed to better mimic the layered structure of native cartilage [192]. PLCL scaffolds with different pore structures and porosity can be obtained by various preparation methods, such as electrospinning, 3D printing, supercritical carbon dioxide foaming, gel extrusion and freeze-drying, so as to better simulate the extracellular matrix structure of different positions and different types of cartilage tissue [193]. Researchers also use PLCL in combination with other materials to give cartilage scaffolds different biological properties: the incorporation of PLCL with natural polysaccharides such as hyaluronic acid and chitosan can improve the hydrophilicity and cartilage regeneration ability of scaffolds [194]; Inorganic materials such as hydroxyapatite(HA) can be added to PLCL to design a scaffold with higher hardness and mechanical strength, which can improve the mechanical properties of the osteochondral repair interface [195]. The combination of PLCL and hydrogel gives consideration to the rigid support of PLCL and the flexible bionic properties of hydrogel, and better simulates the layered structure of cartilage.

**Fig. 10:**
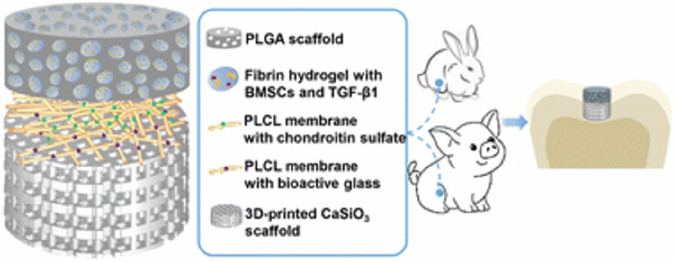
Three-phase bionic BMSCs scaffold loaded with TGF-β promoting the regeneration of hyalene cartilage [196]

Wang et al. used PLCL as the matrix material to construct a three-phase bionic BMSCs scaffold with good osteochondral integration and regeneration ability (Fig. 10). The scaffold was composed of PLGA scaffold loaded with fibrin hydrogel and PLCL fiber membrane added with chondroitin sulfate and bioactive glass, respectively. In addition, BMSC and TGF were loaded into the scaffold to promote the regeneration of chondrocytes. The results of animal models showed that the three-phase scaffold partially degraded after implantation in vivo, significantly promoting the regeneration of hyaline cartilage. The superficial cartilage had good uniformity and elasticity, while the calcified cartilage layer had more mineral components forming and was well integrated with the surrounding tissues. In addition, growth factors such as TGF-β and IGF-1 were added to the scaffold to induce chondrogenic differentiation, promoting chondrocyte proliferation and ECM formation [196]. Jung et al. prepared a cartilage scaffold loaded with TGF-β and Pluronic F127 by gel extrusion. The results showed that this bioactive scaffold containing TGF growth factorscould significantly promote the differentiation of hASCs into chondrocyte phenotype, and promote the formation and integration of cartilage tissue.

PLCL is a pivotal candidate for cartilage tissue engineering scaffolds due to its physicochemical properties, which can be tailored to meet specific tissue demands. Its mechanical performance can be tailored by modulating the lactic acid-to-caprolactone ratio. Scaffolds with diverse pore architectures can be manufactured through various fabrication approaches; additionally, PLCL can be blended with components such as BG and hyaluronic acid to enhance properties including hardness and hydrophilicity, thereby mimicking the structure and functionality of native cartilage. Related composite scaffolds, such as growth factor-loaded three-phase bionic scaffolds, have exhibited promising osteochondral regeneration efficacy in animal studies. Nevertheless, further optimization of the scaffold’s rigidity-flexibility balance is required to withstand the complex biomechanical stresses within joints [197].

### Bone tissue engineering

Bone defects are a very common problem in clinical practice, which can be caused by trauma, infection, surgery and developmental abnormalities. Bone has a strong regenerative and repair ability, which is mainly due to the fact that mesenchymal stem cells in the body can be recruited to the injured area under the action of local inflammatory factors and differentiate into osteoblasts, which further secrete cartilage matrix and gradually ossify and eventually become new bone tissue [198]. At present, the use of biomaterials to promote bone defect repair holds great research potential. The implantation of scaffolds into bone defects can provide local mechanical support, promote the aggregation and growth of mesenchymal stem cells in the injured area, accelerate cell aggregation and osteogenic differentiation in the damaged area, and promote bone repair [199].

**Fig. 11:**
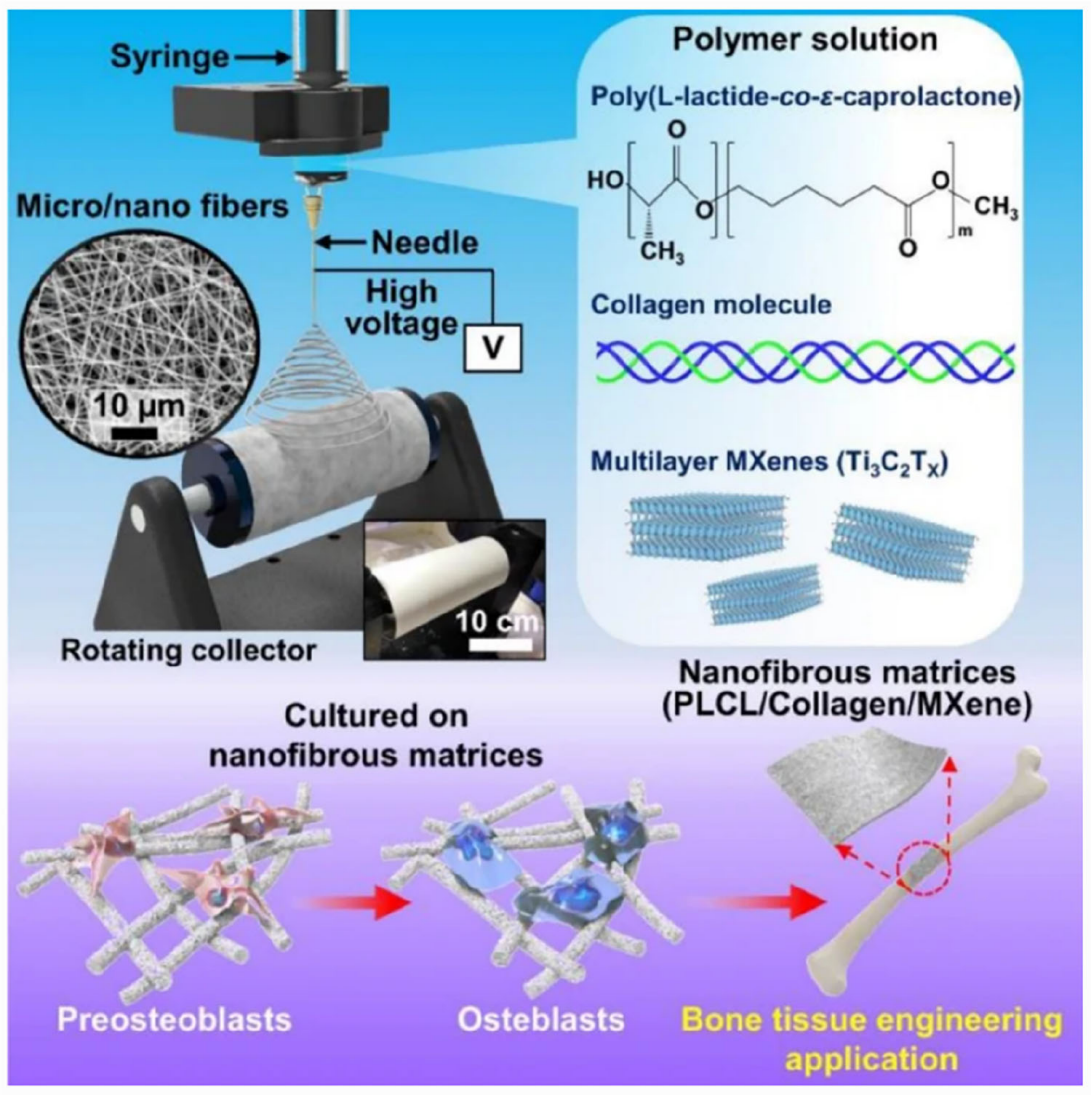
Schematic diagram of ternary MXene-loaded PLCL/collagen nanofibrous scaffolds that promote spontaneous osteogenic differentiation [209]

For bone repair biomaterials, it is necessary to have a high mechanical strength similar to normal bone tissue, to provide sufficient support for bone tissue on both sides of the defect, and to give certain mechanical stimulation to bone tissue at the broken end, to promote bone formation and bone repair, and to have a suitable degradation time to match the speed of bone regeneration and repair and new bone formation [200]. In addition, the scaffold should have a structure and porosity similar to bone trabeculae, and have a surface topography suitable for cell adhesion and growth, promoting cell growth on the surface and inside the scaffold [201]. Finally, the ideal bone repair scaffold should also have biological activity, which can induce the migration and aggregation of mesenchymal stem cells to the injured area and proliferation and differentiation, promote cell ossification and calcium salt deposition, and accelerate the formation of new bone [202]. PLCL has good biocompatibility, and its degradation time is relatively close to the bone tissue regeneration process, which will not affect the growth of new bone due to the non-degradation of the material [203–205]. In addition, PLCL has good processability and can be processed into scaffolds of various shapes and sizes through electrospinning, 3D printing and other technical methods to meet the application of different repair scenarios [206]. de Souza et al. developed an electrospun composite scaffold based on PEG and PLCL, and added calcined and lyophilized silicate chlorinated bioactive glass to enhance the mechanical properties of the scaffold. The results showed that the tensile strength and Young’s modulus of the composite scaffold doped with BG were significantly enhanced. The results of cell experiments showed that the composite scaffold surface can promote cell adhesion and mineralization, which is conducive to the repair of bone defects [207]. Eliton S. de Medeiros et al. prepared a multi-layer bone repair material with a cocoon-inspired bionic structure *via* the SBS technique. Compared with electrospinning, the SBS technique has the advantages of simple operation, low cost, high safety, the ability to fabricate thicker membranes, and broad raw material availability [208]. The fabrication of PLCL bone repair scaffolds *via* the SBS technique is also a highly promising research and development field. Lee et al. doped MXene in PLCL to enhance the osteogenic differentiation induction ability of the scaffold (Fig. 11). Meanwhile, collagen was used to modify the scaffold surface to enhance the cell adhesion ability of the scaffold surface, promote the cell adhesion and growth on the scaffold surface, and promote the spontaneous ossification of MC3T3 [209].

Although PLCL has a certain application in bone tissue engineering, there are some problems when PLCL is applied in bone tissue engineering. There is a big gap between the mechanical strength of PLCL itself and that of normal bone tissue, which is difficult to meet the needs of load-bearing bone repair. Currently, bone regeneration scaffolds based on PLCL are mainly used in the research of non-load-bearing bone repair. This problem requires doping other materials with higher hardness, such as bio-ceramics, into PLCL to improve the overall mechanical strength of scaffolds. In addition, PLCL material itself lacks bone induction ability and cannot provide promoting effect for the osteogenic differentiation and mineralization process of cells, so it needs to be combined with bioactive substances such as BMP to enhance the osteogenic effect of scaffolds.

### Urethral tissue engineering

**Fig. 12:**
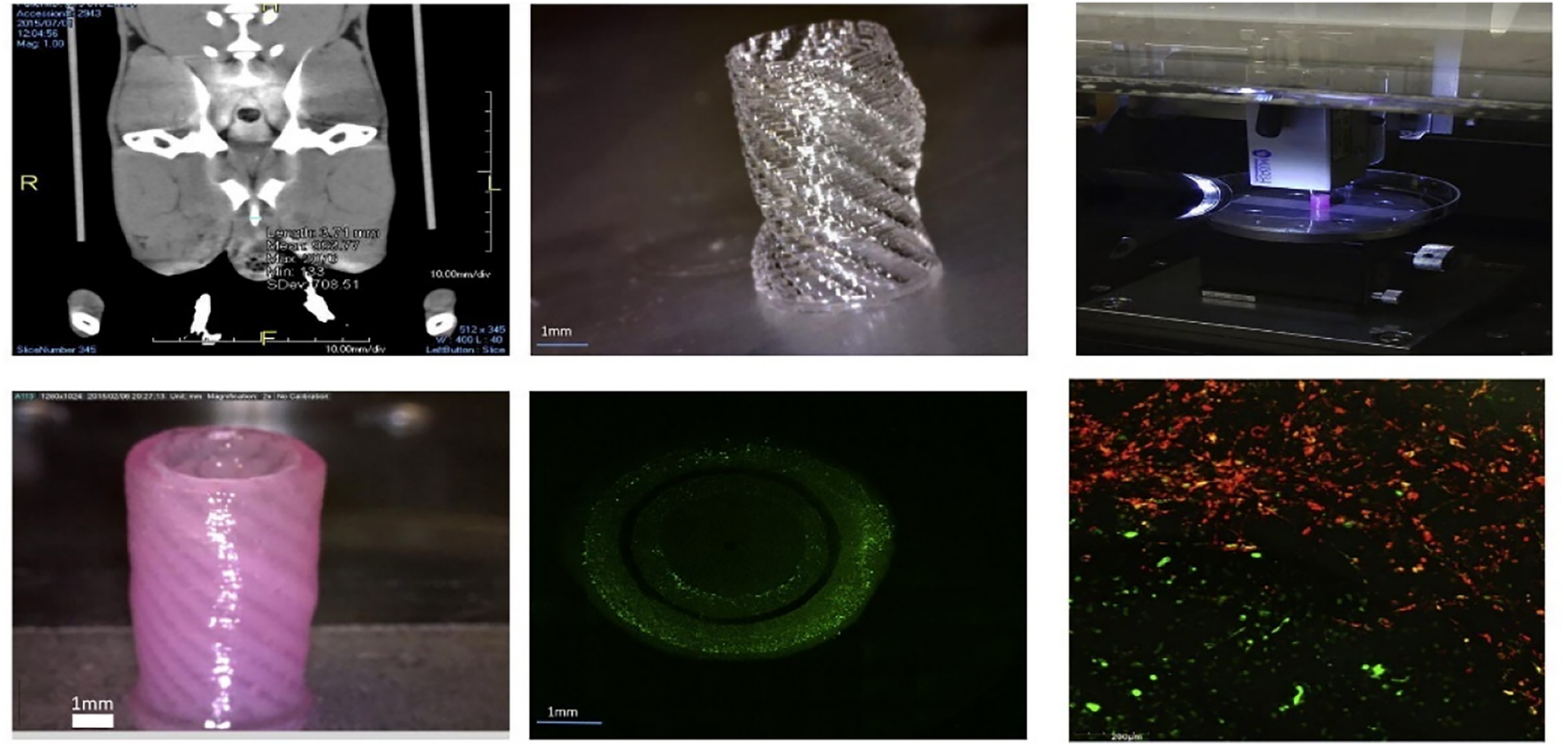
3D-printed PCL/PLCL urethral scaffold loaded with dual autologous cells in fibrin hydrogel [211]

PLCL also finds applications in urologic tissue engineering. PLCL was used to construct urethral stent to repair urethral defect caused by urethral injury. Jiao et al. used electrospinning to construct a PLCL nano-scaffold containing fibrinogen. The results showed that the fibrinogen PLCL composite scaffold can promote the adhesion and growth of epithelial cells, and promote the repair and reconstruction of urethra in rabbit urethral defect model [210]. As shown in Fig. 12, Zhang et al. used 3D printing technology to prepare urethral spiral scaffolds using PLCL and PCL as matrix materials, and loaded urothelial cells and smooth muscle cells on the surface of the scaffolds to construct a bionic scaffold that could simulate the structure of natural urethra tissue. By comparing the mechanical properties of scaffolds prepared with different PCL/PLCL ratios and different printed structures, it was found that the spiral-structured urethral scaffold made from PCL/PLCL (50:50) had mechanical properties closest to those of the native urethra. The cell experiment results showed that the cells on the surface of the scaffold could maintain good biological activity, which provided a reference for the clinical application of 3D printed urethral stent in the future [211].

In addition to replacing the urethra, PLCL has also been studied in bladder regeneration. Feng et al. constructed a coaxial electrospinning biomimetic nanofiber scaffold based on hyaluronic acid and PLCL, which promoted bladder regeneration by regulating the proliferation and migration of smooth muscle cells. The results showed that HA increased the anisotropy and hydrophilicity of the scaffold, and promoted the migration and proliferation of SMC. The results of animal experiments showed that the bladder capacity of rats implanted with composite stents was significantly improved [212]. Shrestha et al. seeded ADSCs onto the surface of PLCL membrane for the repair of bladder injury in rats, and the experimental results showed that the compliance and contractility of the bladder of rats in the PLCL+ADSCs group were significantly improved, and ADSCs were uniformly distributed on the PLCL surface and differentiated into smooth muscle cells. The above studies show that PLCL can act as a matrix carrier of cells to carry out local delivery of cells to the injured area, thus promoting the repair of urinary tissues and organs such as the urethra and bladder [213]. In summary, PLCL’s good biocompatibility, tunable degradation, and excellent processability make it a valuable material for urethral and bladder repair scaffolds. To enhance performance, it is often blended with other materials or loaded with cells. Studies have demonstrated its efficacy in promoting tissue repair with favorable biocompatibility. Nevertheless, the optimization of material blending or processing methodologies is required to align with the mechanical demands of urinary tissues.

### PLCL in other tissue engineering fields

**Fig. 13:**
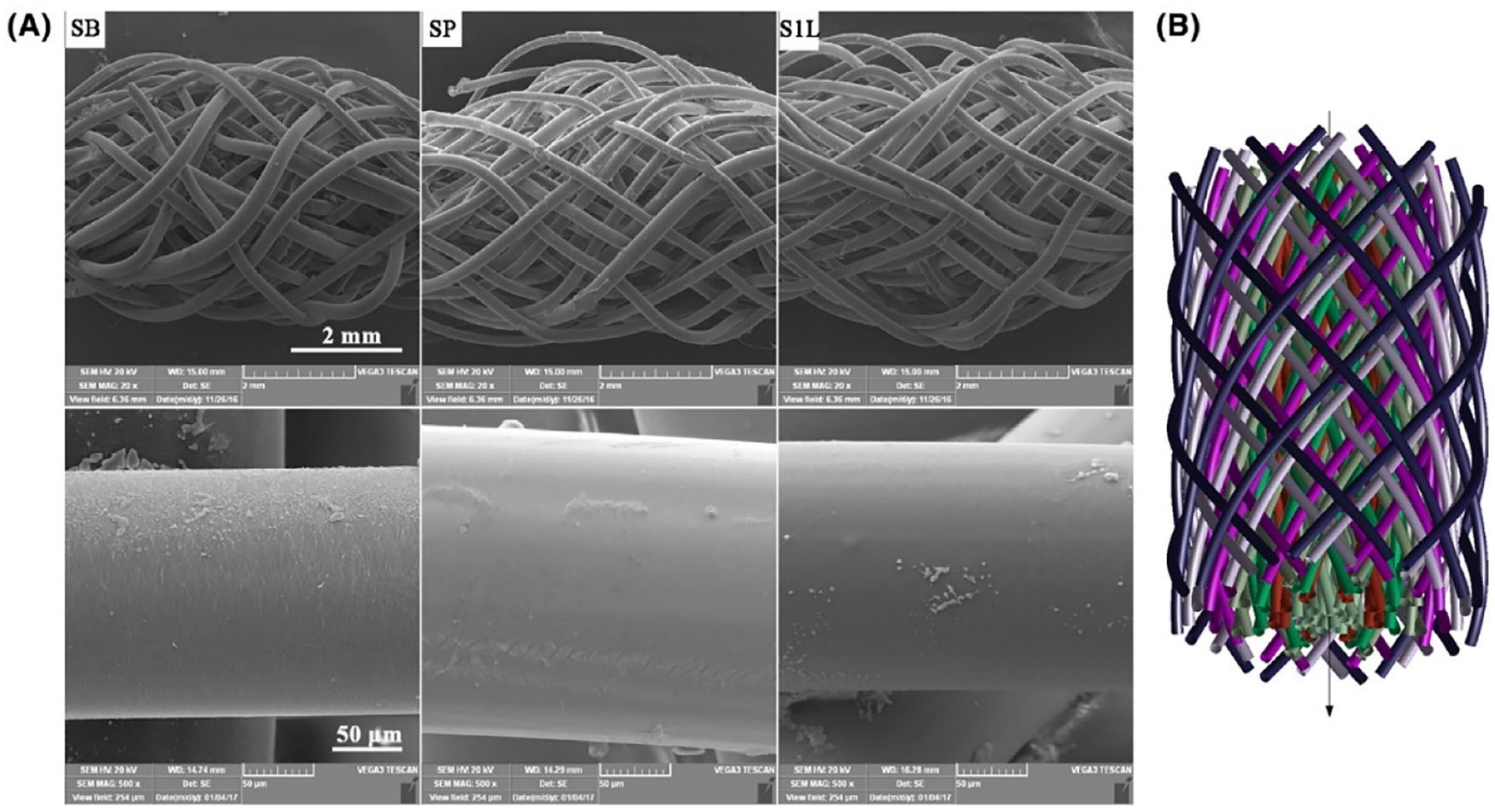
**A** Microscopic morphology of the ligament braided with 16 fibers (SB: PLCL scaffold blank; SP: PLCL-PLL scaffold; S1L: PLCL-PLL/HA-PLL scaffold). **B** Schematic diagram of the global structure of the multi-layer braided scaffold [214]

Beyond the fields discussed, PLCL is also utilized in several other tissue engineering applications. As shown in Fig. 13, Liu et al. developed a PLCL scaffold for ligament tissue engineering. The scaffold is braided with 16 PLCL fibers. The surface of this PLCL scaffold was deposited with a polyelectrolyte membrane of polyl-lysine (PLL) and hyaluronic acid (HA), which could promote the growth of Wharton’s glial mesenchymal stem cells and bone marrow mesenchymal stem cells. The cells formed a spindle-shaped fibrous structure on the surface of the scaffold with good chemotaxis [214]. Jing et al. constructed a PLCL/collagen scaffold packaged with TGF-β to repair tracheal defects in rabbit animal models, and the results showed that TGF-β could be released stably from the scaffold, effectively promote chondrogenesis and differentiation of BMSCs, and promote the repair of tracheal defects [215]. Erdi et al. developed a sprayable PLCL-based tissue adhesive with anti-adhesion and anti-fibrotic properties, which effectively prevented abdominal adhesions post-surgery. This was achieved by selecting PLCL of varying molecular weights and employing SBS technology [85]. Song et al. constructed a corneal scaffold using extracellular matrix and PLCL as matrix materials, and loaded corneal endothelial cells on the scaffold for corneal transplantation. The results showed that CEC had a good adhesion effect on the scaffold, the cell density was similar to that of normal cornea, and the cell phenotype could be maintained. The animal experiment results showed that the ECM-PLCL scaffold could maintain normal corneal characteristics when transplanted into the rabbit eye [216]. Collectively, these studies underscore PLCL’s versatility. Its excellent biocompatibility supports normal cell growth, while its capacity to serve as a carrier for diverse bioactive substances enables its application across a wide range of tissue engineering fields.

## Application of PLCL in other biomedical fields

The applications of PLCL in other tissue engineering and biomedical fields are shown in Table 7.

**Table 7:** Application of PLCL in other in other tissue engineering and biomedical fields

Application fields	Material components	Processing method	Effect	Ref.
Drug screening	PLCL, Gel/PLCL, Col/PLCL	Electrospinning	In vitro construction of cellular tumor spheroids for tumor drug screening.	[219]
Drug screening	rGO/PLCL	Electrospinning	In vitro construction of conduction-consistent cardiac patches for therapeutic drugs screening.	[345]
Controlled drug release	DOX/PLCL/GEL	Coaxial electrospinning	Achieve continuous and stable release of model drug DOX within 20 days.	[220]
Controlled drug release	PDGF-bb /PLCL/DEX	Coaxial electrospinning	A continuous and stable release curve during the subsequent 28-day release cycle after the initial 2-day burst release.	[221]
Stem cell culture	GelMA/PLCL/acellular dermal matrix (ADM)/ Adipose-derived mesenchymal stem cells (ADSCs)	Electrospinning short nanofibers and hydrogel	GelMA-PLCL/ADM Fibers scaffold increased the survival rate of ADSCs within 14 days.	[346]
Stem cell culture	PLCL/GEL/ADSCs/human fibroblast cells (HFCs)	Electrospinning	ADSs/HFCs on the Gel/PLCL nanofiber increased cellular adhesion and proliferation synergistically compared to non-coated plate.	[23]
Abdominal wall defect repair	Sodium alginate/decellularized matrix microgels /PLCL membrane	Electrospinning and hydrogel	An effective mesh material for facilitating the reconstruction of abdominal wall defects.	[347]
Dura repair	HA/PLCL	Electrospinning	The HA/PLCL electrospun membrane was more favorable for fostering dural defect repair and skull regeneration.	[348]
Rotator cuff repair	PGA/PLCL	Electrospinning	In a sheep acute rotator cuff repair model, nanofiber scaffold prevents the recurrence of rotator cuff tears.	[349]
Tendon/Ligament Repair	Polyethylene terephthalate (PET) /PLCL/Gel	Electrospinning	Hybrid nanofibrous composites are integrated with native tendons to guide surrounding tissue ingrowth due to the highly interconnected and porous structure.	[350]
Tracheal defects	PLCL/Col/ TGF-3	Coaxial electrospinning	The tracheal defect model in rabbits was repaired, and re-epithelialization was achieved.	[215]
Prevent abdominal adhesion	PLCL	Solution blow spinning (SBS	Blending 30% (w/v) low-molecular weight PLCL and 70% (w/v) high-molecular weight PLCL in vitro yields an anti-fibrotic, tissue-adhesive polymer sealant.	[85]
Corneal repair	PLCL/ decellularized ECM (UMDM)	Membrane	The PLCL/UMDM membrane is used for corneal endothelial corneal endothelial cell transplantation.	[216]

### Drug screening

At present, screening of anti-tumor drugs is still dominated by two-dimensional cell culture on a plate, but this culture-screening mode lacks cell-cell and cell-extracellular matrix interactions, which cannot effectively evaluate drug reactivity and sensitivity under the conditions of microenvironmental signals in vivo. Three-dimensional cell culture and the construction of multicellular tumor spheres (MCTS) can simulate the extracellular matrix microenvironment in vivo and provide more screening conditions for anticancer drugs [217]. Owing to its excellent processability, PLCL can be fabricated into three-dimensional scaffolds with high porosity and specific surface area *via* techniques such as electrospinning and suspended microsphere fabrication, which can greatly simulate the structure and morphology of extracellular matrix and realize the purpose of drug screening [218]. Ranjbar et al. constructed a PLCL polymerized nanofiber mixed with collagen or gelatin. HT29 colorectal cancer cells formed 3D spherules with uniform morphology and smooth surface on both Col/PLCL and Gel/PLCL nanofibers. The ability to simulate the properties of extracellular matrix in the case of MCTS formation in vitro can be used in the study of cancer drug screening [219]. The anticancer drug screening paradigm dominated by two-dimensional cell culture exhibits inherent limitations, as it cannot replicate the in vivo microenvironmental conditions. Leveraging its superior processability, PLCL can be manufactured into 3D scaffolds with high porosity through electrospinning and comparable techniques, thereby mimicking extracellular matrix architectures. Relevant investigations have verified that tumor cells are capable of forming uniformly structured spheroids on PLCL composite scaffolds, furnishing an optimal in vitro platform for anticancer drug screening.

### Controlled drug release

Due to the controllable and adjustable degradation rate of PLCL, PLCL is also widely used in the study of controlled and sustained drug release. Sustained and controlled release of drugs in vivo can be achieved by mixing drugs directly into PLCL or encapsulating drugs within PLCL microspheres. Liu et al. used PLCL and gelatin as matrix materials and mixed them to prepare core-shell nanofibers capable of loading multiple drugs and factors, which could achieve continuous and stable release of model drug adriamycin within 20 days and avoid in vivo toxicity caused by burst release of doxorubicin hydrochloride (DOX) in the short term [220]. Li et al. prepared an electrospun fiber loaded with PDGF-bb by using dextran and PLCL membranes as ultra-fine core/shell fibers. The results showed that PDGF-bb loaded in DEX/PLCL fibers showed a continuous and stable release curve during the subsequent 28-day release cycle after the initial 2-day burst release. The adhesion growth of vascular smooth muscle cells cultured on the surface of the fibers was promoted [221]. Metecan Erdi et al. fabricated PLCL fibrous membranes *via* SBS, which enables the precise controllable modulation of the release behavior of the anti-inflammatory agent COG133. The drug-loaded PLCL fibrous membranes exhibited excellent anti-adhesion efficacy in animal models of abdominal adhesions [86]. Compared with electrospinning, the fabrication of drug-loaded fibrous membranes *via* SBS exhibits more prominent potential for large-scale production [222]. The SBS process enables the preparation of nanofibrous membranes without the need for high-voltage power supplies or conductive collectors [223]. Thus, SBS demonstrates more competitive advantages in the mass production of PLCL-based drug-loaded fibrous membranes [224].

### Stem cell culture

PLCL can provide a three-dimensional culture environment for the growth of stem cells, promote cell proliferation and differentiation, and can be used in many fields such as tissue engineering and regenerative medicine. PLCL can be prepared into porous scaffolds to simulate extracellular matrix, and three-dimensional scaffolds with different stiffness, elasticity and porosity can be obtained by adjusting the proportion of monomers in PLCL and the preparation process, thereby accommodating different types of stem cells. In addition, the surface modification of PLCL scaffolds can further give the material different surface chemical properties and structural morphology, and promote the differentiation of stem cells in different directions [225, 226]. For example, PLCL combined with HA can be used to promote the differentiation of mesenchymal stem cells towards osteogenesis [227]. The combination of PLCL with collagen and hyaluronic acid is beneficial to the formation of cartilage [228]. The preparation of PLCL into oriented or patterned spinning is beneficial to the differentiation of neural stem cells and the establishment of signal pathways between nerve cells [184]. PLCL is competent to establish a three-dimensional culture milieu for stem cells, modulating cellular proliferation and differentiation to cater to the demands of tissue engineering and related fields. Scaffolds with varied mechanical traits and porous architectures can be achieved via regulating monomer proportions and fabrication protocols. Surface modification or integration with components such as HA and collagen allows PLCL to direct the lineage-specific differentiation of stem cells into osteogenic, chondrogenic and neural phenotypes, thus laying a solid foundation for translational stem cell applications.

## Conclusion and prospect

Owing to its unique combination of biocompatibility, biodegradability, and tunable mechanical properties, PLCL has emerged as a highly promising and widely applicable material in biomedical engineering. Its favorable integration with biological tissues, without eliciting significant immune responses, along with the degradation of its byproducts into natural metabolic intermediates, ensures both safety and long-term efficacy. The ability to precisely tailor the lactide-to-caprolactone ratio and molecular weight provides unparalleled flexibility in tuning the degradation profile and mechanical behavior of PLCL to meet specific tissue engineering demands. Furthermore, through various fabrication techniques and by incorporating drugs, cells, or bioactive factors, PLCL has been successfully applied across diverse fields, including skin wound healing, neural repair, osteochondral regeneration, vascular and urethral graft fabrication, biological meshes, drug delivery, and stem cell culture [1, 18, 196, 225, 229–231].

Despite these advantages, several challenges associated with PLCL must be addressed to advance its biomedical applications. First, although the mechanical properties of PLCL can be modulated, they may still be insufficient for applications under high mechanical loads, such as in load-bearing bone repair or vascular stents. To address this, strategies such as incorporating inorganic nanoparticles (e.g., HA, silica) to form nanocomposites, or blending with other high-strength polymers (e.g., PGA), can be employed to enhance material rigidity and toughness. Second, the inherent hydrophobicity of PLCL can impede cell adhesion, proliferation, and differentiation. This limitation can be mitigated by introducing hydrophilic groups via surface modification or by blending/copolymerizing PLCL with hydrophilic biomaterials such as hyaluronic acid or collagen. Third, while PLCL has been extensively combined with a wide array of bioactive molecules to create multifunctional composites, the in vivo safety profiles of many of these additives require thorough evaluation. This safety concern represents a significant hurdle for the clinical translation and regulatory approval of complex PLCL-based composites. Consequently, when developing PLCL scaffolds for tissue repair, researchers must prioritize the biocompatibility and clinical feasibility of all incorporated components. In conclusion, continued research and development are expected to overcome current limitations and further expand the role of PLCL in advancing biomedical engineering and regenerative medicine.
